# iRGD Peptide as a Tumor-Penetrating Enhancer for Tumor-Targeted Drug Delivery

**DOI:** 10.3390/polym12091906

**Published:** 2020-08-24

**Authors:** Sujin Kang, Sooyeun Lee, Soyeun Park

**Affiliations:** College of Pharmacy, Keimyung University, 1095 Dalgubeoldaero, Dalseo-gu, Daegu 42601, Korea; vegonia1@hanmail.net (S.K.); sylee21@kmu.ac.kr (S.L.)

**Keywords:** iRGD, RGD, integrin, NRP-1, CendR, blood-brain barrier, immunotherapy, tumor-penetrating peptides, tumor microenvironment

## Abstract

The unique structure and physiology of a tumor microenvironment impede intra-tumoral penetration of chemotherapeutic agents. A novel iRGD peptide that exploits the tumor microenvironment can activate integrin-dependent binding to tumor vasculatures and neuropilin-1 (NRP-1)-dependent transport to tumor tissues. Recent studies have focused on its dual-targeting ability to achieve enhanced penetration of chemotherapeutics for the efficient eradication of cancer cells. Both the covalent conjugation and the co-administration of iRGD with chemotherapeutic agents and engineered delivery vehicles have been explored. Interestingly, the iRGD-mediated drug delivery also enhances penetration through the blood–brain barrier (BBB). Recent studies have shown its synergistic effect with BBB disruptive techniques. The efficacy of immunotherapy involving immune checkpoint blockades has also been amplified by using iRGD as a targeting moiety. In this review, we presented the recent advances in iRGD technology, focusing on cancer treatment modalities, including the current clinical trials using iRGD. The iRGD-mediated nano-carrier system could serve as a promising strategy in drug delivery to the deeper tumor regions, and be combined with various therapeutic interventions due to its novel targeting ability.

## 1. Introduction

Although advancements in therapeutic techniques have increased the life expectancy of cancer patients, the growing rate of cancer incidence still remains a matter of concern [1,2]. The standard treatment for various solid tumors includes chemotherapy and radiotherapy, following the surgical removal of the tumor mass. Remarkable advancements in chemotherapy have not only reduced cancer mortality, but also improved the quality of life for cancer survivors. Nevertheless, there are still unresolved challenges in chemotherapy, including poor drug penetration, chemotherapeutic resistance, short half-life, and side effects owing to cytotoxicity in healthy cells [3,4,5].

Conventional chemotherapeutic agents need to access the tumor tissues in order to destroy tumor cells. Chemotherapeutic agents usually penetrate 3–5 times the diameter of cells in solid tumors. Nonetheless, their concentration in deep regions of tumor beyond this penetration depth is significantly lower than the intended concentration, thus leading to tragic relapse or acquisition of drug resistance in cancer treatment [6]. In addition, the unique structure and physiology of the tumor microenvironment hamper the penetration of chemotherapeutic agents. Tumors are heterogeneous cellular entities composed of cancer cells and the surrounding tissues, including stromal cells, immune cells, mesenchymal stem cells, and extracellular matrix (ECM) [7,8]. Most solid tumors originate from epithelial cells, whose morphology is characterized by specialized structures at the boundaries between neighboring cells for cell–cell adhesion [9]. Junction proteins, such as desmoglein 2 (DSG2) and E-cadherin, have been found to be up-regulated in malignant tumor cells [10,11]. The enhanced adhesion between cells provides a physical barrier against intercellular transport of molecules, including chemotherapeutic drugs [9]. During tumor progression, there is an overproduction of the ECM constituents, such as collagens and fibronectins, which gradually build up a dense network. Studies have shown that the increased density of ECM in tumors results in the low penetration of anti-cancer drugs [12,13,14]. Particularly, the penetration of paclitaxel in tumors was linearly reduced as the cell density of the tumor increased [15]. Clinicians often detect cancer as a massive lump by palpation [16]. From a mechano-biological point of view, the existence of this massive lump is a clinical proof that tumor tissues are mechanically stiffer than healthy tissues, which is due to the increased density of the ECM [17,18]. The enhanced stiffness of ECM has been considered to intensify the interstitial fluid pressure (IFP), thus impeding the effective penetration of anti-cancer drugs into the solid tumor [19,20,21].

In general, the tumor vasculature possesses an immature nature owing to its rapid and aggressive growth and insufficient smooth muscle cells, thus displaying a discontinuous endothelial cell lining. The structural conditions of solid tumors such as leaky vasculature lead to the enhanced permeability and retention (EPR) effect that permits macromolecules to enter the tumor interstitial space, while the suppressed lymphatic filtration allows them to be retained in tumor tissues [22,23,24]. This passive tumor targeting explains how nano-carriers of sizes 20–200 nm extravasate and accumulate inside the interstitial space [25]. The penetration of a drug into the tumor via this passive delivery relies extensively on random chances allowed by tumor vascularization and angiogenesis [26]. Additionally, the magnitude of the EPR effect depends on the tumor type and heterogeneity [27,28,29]. Moreover, the high IFP in solid tumors disrupts the effective uptake and distribution of drugs through passive targeting [21]. In contrast, active tumor targeting utilizes the high binding affinity between ligands and specific receptors expressed on target sites to enhance the penetration of drugs into the target sites [30,31]. The binding affinity to receptors specifically expressed in the tumor endothelial cells controls the cellular uptake of the nano-carriers [32]. Remarkably, recent reports have indicated that the delivery efficiency of nanoparticles in solid tumors is currently as low as ~0.7% of the administered dose, despite all the efforts to enhance the drug uptake [33,34].

Many studies have investigated whether the modulation of epithelial junctions and ECM structures can improve the intra-tumoral penetration of anti-cancer drugs beyond the passive targeting ability relying on the EPR effect. Degradation of ECM proteins or the reduction in their expression is a reliable approach for improving the penetration and dispersion of anti-cancer drugs [35]. As an example, relaxin, which binds with leucine-rich repeat-containing G protein receptors (LGR) on tumor tissues [36], was found to effectively break down ECM components and enhance the expression of matrix metalloproteinases [37]. Cancer virotherapy studies showed that relaxin can improve the distribution and penetration of oncolytic adenovirus in tumors [38]. However, its effect on tumor progression remains controversial. Overexpression of relaxin resulted in the increase in prostate tumor growth and angiogenesis [39]. Furthermore, breast cancer cells exposed to relaxin, even for a short duration, showed an increase in invasiveness [40]. As an alternative, the opening or modification of the epithelial junction enables anti-cancer drugs to penetrate tissue barriers. The junction opener-1 (JO-1), a recombinant protein derived from the adenovirus serotype 3, binds and cleaves DSG2, thereby triggering the epithelial mesenchymal transition (EMT)-like signaling, leading to transient opening of epithelial junctions in tumors [41,42,43]. The co-administration of JO-1 opened tight junctions in epithelial tumors and increased the therapeutic efficacy of several chemotherapy drugs [44]. Recent studies evaluating the efficacy and safety of JO-1 did not report any blood or tissue complications, nor expressed concern on overall health and behavior of the patient. However, the activation of EMT by JO-1 binding to DSG2 might facilitate tumor metastasis, although no direct evidence from a long-term follow-up has been found [44]. Its possible immunogenicity is another issue.

Chemotherapeutics targeting tumor vasculature can directly bind with the blood vessels or vascular endothelial cells, and have less possibility to cause drug resistance due to high genetic stability of endothelial cells [45]. iRGD, a novel cyclic peptide composed of 9-amino acids including an Arg-Gly-Asp (RGD) motif, has a high binding affinity to αvβ3 and αvβ5 integrins abundant in tumor vasculatures. iRGD-conjugated and co-administrated drugs showed enhanced distribution throughout the extravascular tumor parenchyma. Compared with RGD, the tumor targeting ability of iRGD is more intensified because iRGD can specifically bind to integrins and neuropilin-1 (NRP-1) receptors that are overexpressed on various tumors. The cleaved form of iRGD binds to NRP-1, and subsequently triggers NRP-1-dependent endocytosis, thus resulting in enhanced tumor penetration. iRGD shows attractive advantages in delivery systems, including low toxicity to normal cells [46], easy and low-cost synthesis [47], and targeted release [48,49].

At present, the iRGD peptide has attracted significant attention as a promising delivery moiety for improving the intra-tumoral penetration of chemotherapeutic agents through angiogenetic vessels. In this review, we will present and summarize the literature describing the underlying mechanism of the iRGD-mediated tumor-targeting in the milieu of tumor stroma. First, we describe its two major implementation strategies, i.e., co-administration approach and the targeting moiety approach. Then, we will highlight its recent applications in immunotherapy and clinical trials. Lastly, we will focus on the role of iRGD-mediated transport in drug delivery to the brain. Collectively, it is expected that application of iRGD can be incorporated into newly developed treatment modalities, such as immunotherapy, for improving the overall chemotherapeutic efficacy. Advantages of iRGD peptide will not only allow the resolution of problems present in chemotherapeutic agents, such as poor drug penetration and side effects, but also improve the pharmacokinetic properties of the drug delivery system.

## 2. iRGD Peptide

### 2.1. Discovery of iRGD Using Phage Display Screening

The RGD peptide is a representative example of vasculature-targeting peptides, employed for the delivery of anti-cancer drugs or imaging agents [50,51]. The RGD peptide displays a specific binding affinity to αvβ3 and αvβ5 integrins. The novel cyclic peptide iRGD, containing the RGD motif, was identified by Ruoslahti and colleagues through phage display screening [52]. To isolate peptides with high affinity to tumor tissues, they utilized the cyclic CX7C (C = cysteine; X = any amino acid) peptide library displayed on the T7 phage [53]. Tumor-related phages were selected through three rounds of ex vivo and in vivo phage screenings. The newly obtained phage pool showed 200–400 times stronger binding affinity to tumor-derived cell suspensions than the original library. In addition, the binding affinity to tumor cell suspensions was five times stronger than that to cell suspensions derived from normal bones. Random clones were selected from the phage pool for sequencing. The three peptides containing the RGD motif, i.e., CRGDKGPDC, CRGDRGPDC, and CRGDKGPEC, were identified in the selected pool. The phage displaying CRGDKGPDC peptide was bound and internalized to primary prostrate carcinoma (PPC1) cells at 4 °C and 37 °C, respectively. The internalized peptide was named “iRGD”. In general, iRGD refers to the cyclic 9-amino peptides with sequences CRGD[K/R]GP[D/E]C [52].

### 2.2. Dual Targeting Mechanism of iRGD in Tumor Treatment

**Figure 1:**
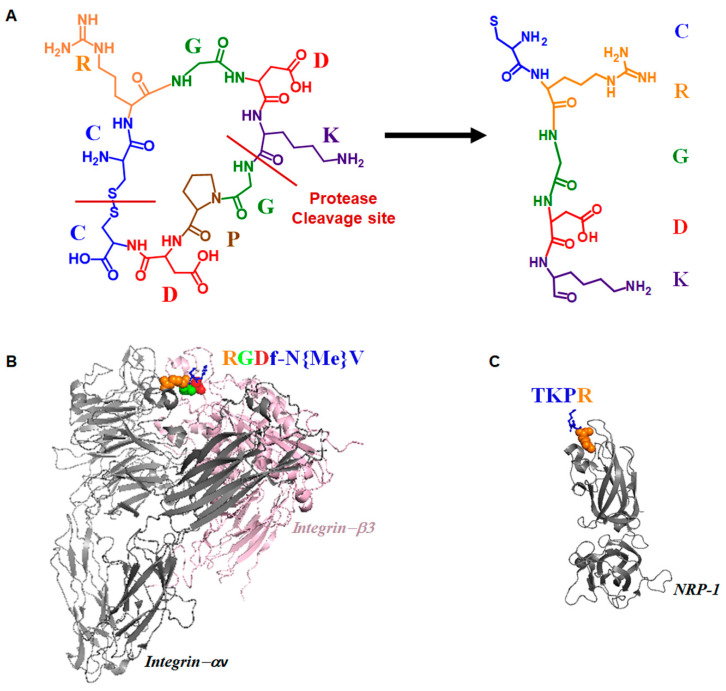
Illustration of cleavage process and the binding events of cyclic iRGD [CRGDKGPDC] peptide. (**A**) iRGD is proteolytically cleaved to expose the cryptic C-end Rule (CendR) motif. The cleavage sites are indicated with red lines. (**B**) iRGD binds with integrins. As an example, we present the structure of integrin αvβ3 complexed with cyclo (RGDf-NV) reproduced from PDB code 1I5G [68]. (**C**) After cleavage, the arginine or lysine of the CendR motif binds to the b1 domain of neuropilin-1 (NRP-1). The presented structure was reproduced from PDB code 2ORZ showing the binding event of NRP-1 and Tuftsin mediated by arginine moiety [55,69,70]. The spheres represent the binding residues of polypeptides and blue ball-sticks represent non-binding residues. The color indicates the corresponding sequence of peptides.

iRGD is a prominent enhancer that enables anti-cancer drugs to penetrate tumorigenic blood vessels. It enhances the permeability to tumor vessels, triggers internalization, and penetrates deep into tumor tissues, thus resulting in improving the diagnostic sensitivity and therapeutic efficacy. The disulfide-based cyclic iRGD peptide has flexible structural conformations [52,54]. The peptide has three distinct sites: a tumor homing motif, a C-end Rule (CendR) penetration motif, and a proteolytic cleavage recognition site [52,55]. The cleavage process and structural illustration of the binding events of iRGD are presented in Figure 1.

The integrin-dependent binding is the first step in iRGD’s tumor-targeting process. Integrin is a heterodimeric transmembrane glycoprotein consisting of α- and β-subunits and is known to mediate cellular adhesions to ECM [56,57]. Integrin expression is strongly correlated with the cell type and microenvironment [58,59]. Eight different integrin heterodimers, viz., αvβ1, αvβ3, αvβ5, αvβ6, αvβ8, α5vβ1, α8β1, and αIIbβ3, recognize the RGD motif within ECM proteins [60]. Ruoslahti and colleagues first identified αvβ3 [61], which is overexpressed in various cancers, including gastric cancer [62], glioma [63], non-small cell lung cancer [64], pancreatic cancer [65], and prostate cancer [66]. Later, αvβ5 was identified as an important regulator in these cancer types [62,64,66,67].

**Figure 2:**
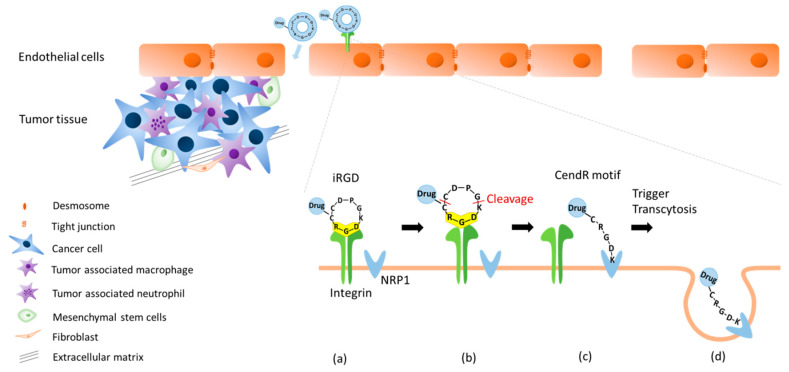
Schematic representation of the iRGD-activated tumor-targeting for nano-carrier delivery in solid tumors. Tumors are composed of cancer cells surrounded by an extracellular matrix (ECM) and stromal cells, including tumor-associated macrophages, tumor-associated neutrophils, mesenchymal stem cells, and fibroblasts. (**a**) First, the iRGD (CRGD[K/R]GP[D/E]C) peptide binds to αvβ3 and αvβ5 integrins on tumorigenic endothelial cells. (**b**) iRGD is proteolytically cleaved to produce CRGD/K and expose the CendR motif at the C-terminus. (**c**) Then, CendR binds to NRP-1 to trigger the penetration into the tumor tissue. (**d**) Finally, internalization into tumor sites via endocytosis is achieved.

After binding to integrin, iRGD is proteolytically cleaved to produce CRGD/K and expose the cryptic CendR motif (R/KXXR/K) at the C-terminus arginine or lysine residues. Then, the CendR motif binds to NRP-1 and subsequently triggers an active endocytosis to internalize iRGD [55]. Through this mechanism, the drugs conjugated or co-administered with iRGD can effectively penetrate deeply into the tumor sites (Figure 2) [49].

Similar to RGD, the iRGD peptide has a specific binding affinity to αvβ3 and αvβ5 integrins in the nanomolar range [71,72]. The CRGDK fragment cleaved by a protease showed an approximately 50- to 150-fold higher binding affinity to NRP-1 than integrins. It has been reported that the proteolytic cleavage results in a decrease in the rigidity of the CRGDK fragment, thus increasing its binding affinity to NRP-1 [52]. The shift in CRGDK from integrins to NRP-1 activates endocytosis, consequently leading to enhanced penetration [73,74]. This shift in the binding affinity enables iRGD peptides to be spread throughout the interstitium. Conversely, RGD peptides only bind to integrins, but not to NRP-1, and are thus accumulated only inside and/or around tumor vessels. Notably, the expression of αv integrins is largely restricted in specific types of tumors, whereas that of NRP-1 is enhanced in many tumor types. Compared with RGD peptides, the iRGD peptide is an efficient targeted drug delivery moiety with enhanced penetration due to CendR- and NRP-1-dependent penetration [52].

NRP-1, a transmembrane glycoprotein, contains a short transmembrane domain. It was first described as a neuronal adhesion molecule in the nervous system [75]. NRP-1 is essential for neural crest migration and axon guidance during neuronal development [76,77]. Moreover, NRP-1 has been implicated in angiogenesis, as its overexpression in mice embryos shows that it is involved in the formation of blood vessels, excess capillaries, and hemorrhaging [78,79]. NRP-1 is also recognized as a co-receptor that enhances the binding of the vascular endothelial growth factor (VEGF)-A to the VEGF receptor [80]. Its upregulation triggers the activation of VEGF signals involved in tumor angiogenesis [81]. NRP-1 is found to be overexpressed in many tumors, including breast cancer [82], melanoma [80], and glioblastoma [54], indicating that NRP-1 plays a critical role in tumor progression. The correlation of NRP-1 overexpression and cancers was analyzed using meta-analysis over 15 studies involving a total of 2049 patients [83]. It was observed that median NRP-1 expression was 59.54%. Reports have established that NRP-1 overexpression varies by cancer type. It was 30%, 51.74%, 61.99%, and 56.76% in tongue cancer, pancreatic ductal adenocarcinoma (PDAC), breast cancer, and glioma, respectively [83]. There was a difference in median survival of patients with high and low NRP-1 expression; it was shown as 16.7 (0.5–46.6) and 34.9 (5.6–94.3) months, respectively. The low survival rate was highly correlated with NRP-1 overexpression [84]. Furthermore, the expression of NRP-1 had patient-to-patient variations, which complicates the simple prediction of the treatment outcomes. For example, patients with no gland formation (nGF) types of gastric cancer suffered from poor prognosis [85]. The study suggested that 70.8% of gastric cancer cases with nGF types showed high expression of NRP-1, while only 65.6% (84/128) of cases with the gland formation type had its overexpression.

The interaction between iRGD and NRP-1 overexpressed in endothelium cells is effectively utilized to penetrate tumor cells in targeted drug delivery systems [54]. For example, the co-administration of the iRGD peptide elevated the anti-cancer efficacy of gemcitabine in a murine pancreatic cancer model which showed an overexpression of NRP-1. These results suggest that the co-administration of iRGD peptide would be beneficial for patients with NRP-1 overexpressing tumors [84].

### 2.3. Active Targeting of iRGD Peptide to Exploit the Tumor Microenvironment

In adults, angiogenesis is tightly controlled, and normal vasculature becomes largely quiescent. In contrast, the formation of tumor vasculature is abnormally regulated and is continuously stimulated by a variety of factors, such as hypoxia, high IFP, fibrosis, inflammation, and acidity [86,87,88]. Particularly, the shortage of oxygen occurs at places distant from blood vessels, causing hypoxic conditions, and triggers the activation of the VEGF and the platelet derived growth factor [89]. The activated endothelial cells induce the up-regulation of integrin αvβ3 [90], which recognizes the ECM proteins and promotes endothelial cell migration [91]. They also trigger the activation of proteolytic enzymes, which disrupts the basal membrane. The subsequent apoptosis of endothelial cells provokes the urgent formation and dramatic remodeling of the vessel lumens, resulting in immature and leaky vasculatures. These vasculatures suffer from fenestrations, irregular blood flow, inadequate lymphatic drainage, and the loss of smooth-muscle layer and pericytes [92]. This poorly organized vasculature is a route for passive transport of nano-carriers. Concomitantly, active targeting to cancer cells or tumoral endothelium aim to improve the transport of anti-cancer drugs. Remarkably, the active targeting strategy using iRGD, containing the tumor-homing CendR motif, resolves the issues concerning the limited penetration of drugs. The active targeting strategy often utilizes the biological interaction between ligands and receptors specifically expressed on target sites. A study using fluorescein (FAM)-labeled peptides demonstrated that the conjugation of iRGD enables the drugs to be effectively distributed at the tumor parenchyma, whereas FAM-inactive controls were not found within or around the tumor. In addition, the anti-tumor effect of iRGD in drug delivery was stronger compared with that of conventional RGD [93]. Although conventional RGD peptides were only found in and around the tumor vessels, cumulative evidence suggests that iRGD-containing cryptic CendR motif readily entered the tumor parenchyma [52,94,95].

In an earlier study, Wang et al. reported that the iRGD-conjugated doxorubicin-polymeric nanoparticles (NPs) effectively penetrated tumors, and exhibited an anti-angiogenetic effect [93]. Additionally, experiments using iRGD modified liposomes (R-LP) encapsulating elF3i shRNA (R-LP/shelF3i) revealed highly suppressed tumor migration and invasion. Inhibition of pulmonary metastasis, angiogenesis, and tumor proliferation was observed in R-LP/shelF3i-injected mouse model [96]. In addition, the study confirmed that the iRGD-conjugated pigment epithelium-derived factor (PEDF)-DNA-loaded liposomes (R-LP/PEDF) exerted anti-invasion and anti-migratory activities in colorectal cancer cells, and extended the survival time in a mouse model [97]. The capability of anti-cancer drugs to reach the deep regions of tumor with sufficient concentration is essential for achieving therapeutic efficacy. As discussed above, use of the iRGD peptide is a promising strategy that achieves enhanced penetration and effective delivery of anti-cancer drugs, thus eradicating cancer cells in the deeper regions of tumors, which are inaccessible to conventional chemotherapeutic agents.

## 3. Applications of iRGD in Cancer Therapy

### 3.1. Implementation Strategies in iRGD Technology

As summarized in Table 1 and Table 2, there are two strategies for using the iRGD technology to enhance chemotherapeutic efficacy: its covalent conjugation, and the co-administration with anti-cancer compounds or drug delivery vehicles. The conjugation strategy refers to the method in which the iRGD peptide is chemically or physically bonded to anti-cancer drugs, or encapsulated in delivery vehicles with anti-cancer drugs. To conjugate iRGD with anti-cancer drugs, maleimide-thiol reaction [98], Michael addition of acryloyl-amine reaction [99], alkyneazide click reaction [100], or amidation of carboxyl-amine reaction [101] were utilized [102]. This conjugation strategy has been implemented in many delivery vehicles, including liposomes, micelles, and polymeric NPs (Table 1). Meanwhile, some studies simply applied the iRGD peptide as a co-administrated agent with chemotherapeutics, without any devised design for advanced conjugation; these methods are summarized in Table 2. Both strategies have equally yielded significant improvements in drug penetration and chemotherapeutic efficacy [103,104]. These studies have also reported similar advantages, including targeted delivery and reduced toxicity of anti-cancer drugs. To date, there has been no report on the advantage of one strategy over the other.

Liposomes display excellent properties as a drug delivery vehicle, including sustained release and loading capability for both hydrophobic and hydrophilic drugs [105,106]. The surface conjugation of iRGD to liposomes confers the additional advantage of tumor penetration as shown in Table 1. For example, Song et al. reported that the liposomes loaded with iRGD-conjugated doxorubicin enhance the anti-tumor activity in 4T1 breast cancer cells and the depletion efficiency of tumor-associated macrophages (TAMs) as compared with doxorubicin-loaded liposomes [107]. In addition, studies using iRGD-modified indocyanine green (ICG) liposomes (iRGD-ICG-LPs) reported an increase in tumor inhibitory effect through photothermal therapy (PTT)/ photodynamic therapy (PDT) effects. Evidently, iRGD-ICG-LPs provided the sensitive detection of 4T1 breast tumor through near-infrared (NIR) fluorescence imaging to enhance their accumulation in tumor [98]. Bao et al. have also demonstrated that the iRGD-conjugated pigment PEDF-DNA-loaded liposomes showed enhanced anti-metastatic, apoptotic, and cytotoxic activities in colorectal cancer cells, and extended survival time in vivo [97].

A number of studies also investigated whether the iRGD-conjugated micelles were effective vehicles to deliver chemotherapeutics for treating various cancers, including prostate, pancreatic, breast, cervical, and brain cancer, using iRGD-conjugated micelles (Table 1). Studies included taxen- and platinum-based chemotherapeutics. Sugahara et al. observed the enhanced tumor penetration in pancreatic ductal adenocarcinoma-bearing mice after injection with iRGD-conjugated micelles [52]. It was reported that iRGD-linked polymeric micelles efficiently entered cells via endocytosis [108]. The enhanced drug accumulation in glioblastoma and its effective transportation through the blood–brain barrier (BBB) were reported from the studies using iRGD conjugated micelles [103,104].

Numerous studies have evaluated the effect of iRGD conjugation to polymeric NPs (Table 1). Polymeric NPs are often utilized to decrease the toxicity, and to improve the solubilization and efficacy of chemotherapeutics. Lipid-polymeric NPs solicit more advantages by using biodegradable polymers and biomimetic lipids [109]. For example, Zhu et al. found that the iRGD conjugation on paclitaxel-loaded Poly(ε-caprolactone)-Poly(N-vinylpyrrolidone) (PCL-PVP) polymeric NPs enhances the drug accumulation and penetration at tumor sites. Additionally, H22 tumor-bearing mice with the iRGD conjugation on paclitaxel-loaded PCL-PVP polymer showed a reduction in tumor growth and survival extension [110]. Gao et al. showed that the iRGD-modified lipid–polymer hybrid NPs loaded with isoliquiritigenin (ISL-iRGD NPs) inhibit the tumor growth in tumor-bearing models. ISL-iRGD NPs also induced enhanced apoptosis in breast cancer cells [111]. The iRGD-conjugated polymeric NPs were also implemented to improve the efficacy of boron neutron capture therapy, which has been limited by the difficulty of targeted delivery to tumors. The study revealed increases in the uptake of both doxorubicin and boron in A549 cells [112].

Additionally, several studies have investigated the conjugation of iRGD to hydrogels (Table 1). Su et al. observed that doxorubicin-encapsulated and iRGD-conjugated nanogels, with thermo- and pH- responsive properties, facilitated controlled drug release in B16 tumor cells. Cellular uptake of doxorubicin-encapsulated iRGD-conjugated nanogels was also observed in B16 melanoma cells, with the alleviation of side effects caused by doxorubicin [125]. The iRGD-conjugated hydrogels of gambogic acid nanoparticles (GA-NPs) promoted the antitumor activity due to the enhanced penetration into tumor sites, ultimately leading to reduced tumor volume and a distinct anti-tumor effect [139].

Similar to the conjugation approach, the co-administration of iRGD with therapeutic drugs also resulted in improved drug efficacy (Table 2). Sugahara et al. emphasized that the tumor-penetrating effect does not require the conjugation of peptides. Instead, a simple co-administration with iRGD improves drug efficacy, such as that of doxorubicin and nab-paclitaxel (Abraxane^®^). The iRGD moiety, via binding of the CendR motif and NRP-1, activates a bulk transport process that sweeps along compounds or therapeutic drugs present in blood [54,139]. Despite this report, there have been continuous attempts to maximize its effect by devising innovative vehicles through conjugation techniques. Nevertheless, it is worthwhile to understand the improvements in chemotherapeutic efficacy resulting from the simple co-administration of iRGD, as summarized in Table 2.

Some studies investigated whether the co-administration of iRGD with drug delivery systems can improve chemotherapeutic efficacy. For example, Zhong et al. showed that the co-administration of iRGD with the paclitaxel-loaded poly (lactic-co-glycolic acid) (PLGA) nanoparticle facilitates drug accumulation in tumors, and improves the antitumor effects when compared with the paclitaxel-loaded PLGA without iRGD co-administration [129]. The co-administration of iRGD with irinotecan-loaded silicasome was also reported to activate the NRP-1-mediated transcytosis transport pathway in pancreatic ductal adenocarcinoma, supporting the notion that iRGD can improve the efficacy of irinotecan-based silicasome [131]. Deng et al. also showed that the combination of iRGD with self-assembled amphiphilic block copolymer NPs (HA-PLA) facilitates the drug distribution and tumor penetration in lungs, and consequently inhibits metastatic breast cancer [132].

In addition, many studies have investigated whether the simple co-administration without associating with any delivery vehicle can lead to enhanced drug penetration and efficacy. The simple co-administration of iRGD with chemotherapeutics has been evaluated in numerous studies. TNF-α-related apoptosis inducing ligand (TRAIL) therapy is often limited by the drug-resistance of cancer cells, although TRAIL is an attractive anticancer agent owing to its selective targeting of cancer cells. The effect of the recombinant TRAIL against cancer cells was examined to overcome the inhibition of TRAIL-resistance by combining the therapeutic effects of sorafenib and iRGD [138]. When sorafenib and iRGD were co-administrated with the recombinant human izTRAIL, the anti-cancer effect of izTRAIL was observed to be enhanced in HT-1080 fibrosarcoma-bearing mice. In addition, the co-administration of iRGD with gemcitabine induced the effective apoptosis in A549 xenograft model compared with the solo administration of gemcitabine [136]. In the A549 xenograft model, the co-administration of gemcitabine and iRGD yielded decreases in tumor volume and growth than the administration of gemcitabine alone. Additionally, the co-administration of iRGD with the membrane-active peptide HPRP-A1 augmented the reactive oxygen species (ROS) production and mitochondrial depolarization in A549 cells. The combinatorial treatment using iRGD and HPRP-A1 has been shown to be more effective in terms of tumor penetration and accumulation than HPRP-A1 alone [48].

### 3.2. Recent Clinical Trials with Co-Administration of iRGD in Pancreatic Cancer

PDAC is a virulent cancer, and its aggressive biology contributes to a poor prognosis in pancreatic cancer patients. Surveillance, Epidemiology, and End Results (SEER) statistics during 1974–2014 indicated that PDAC incidence and mortality rates have continually increased in the US for decades [140]. Unlike other cancers, 5-year relative survival rate show little improvement [141]. As per earlier reports, pancreatic cancer is the forth-leading cause of cancer death [140], and may probably become the second leading cause in the US by 2030 [142].

PDAC does not show any obvious symptoms in the early stage, except abdominal pain, indigestion, and weight loss due to anorexia. This silence at the early stages makes it difficult to diagnose early enough to perform surgical resection. Furthermore, there is a lack of markers for early detection. The location of tumor, i.e., retroperitoneum, and the systemic effects of the disease also limit the choices for local treatment. Reports indicate that chemotherapy may produce a small benefit [143]. Gemcitabine is the first-line standard treatment for PDAC. Many studies have attempted to improve the effects of gemcitabine using other chemotherapeutic agents, such as nab-paclitaxel, oxaliplatin, fluorouracil, and leucovorin [144]. In particular, nab-paclitaxel increased the intra-tumoral concentration of gemcitabine [145]. A synergistic effect with nab-paclitaxel was observed in murine models with pancreatic cancer [145,146]. The co-administration of gemcitabine and nab-paclitaxel significantly extended the overall survival and progression-free survival in patients with advanced pancreatic cancer [28]. Thus, the combination therapy of gemcitabine with nab-paclitaxel has been suggested as a standard therapy for pancreatic cancer. Nevertheless, the outcome of chemotherapeutic treatments on pancreatic cancer remains unsatisfactory. The failure of chemotherapeutic treatment of PDAC is closely related with aggressive cellular properties and the acquisition of chemotherapeutic resistance. Stromal proliferation through non-cell-autonomous action, decreases in vascular density, and inhibition of immunosuppression contributes to chemotherapeutic resistance. As the basic properties of PDAC are revealed, recent clinical trials have focused on the specific targeting of tumor stroma [143]. The iRGD peptide has been employed for the treatment of pancreatic cancer. NRP-1 is aberrantly upregulated in PDAC [147,148,149], and is thus an attractive target for deterring the progression of PDAC [150]. The combination of the iRGD peptide with silicasome-based chemotherapy was shown to trigger NRP-1–mediated transport in PDAC, resulting in improved survival rates and reduced metastatic progression [131]. The co-administration of the iRGD peptide with gemcitabine resulted in effective drug accumulation and tumor reduction in patient-derived PDAC xenografts with NRP-1 overexpression [84]. In addition, the combination therapy of iRGD with nab-paclitaxel slightly reduced the tumor growth of BT474 breast tumors [54].

Presently, there is an ongoing Phase 1 clinical trial study to evaluate the safety and preliminary efficacy of CEND-1 in combination with gemcitabine and nab-paclitaxel (CEND1-001, Clinical trial reference NCT03517176) [151]. The study was designed as an open-label and multi-centered trial. For dose escalation, the safety of ascending dose levels of CEND-1 was evaluated. Initially, four different doses of monotherapy CEND-1 have been administered for one week. Subsequently, its combination therapy with gemcitabine and nab-paclitaxel was performed for 28 days. The co-administration of CEND-1 peptide with gemcitabine and nab-paclitaxel is expected to improve the treatment efficacy for pancreatic cancer patients.

### 3.3. iRGD Application with Immunotherapy

Cancer immunotherapy focuses on the activation of immune surveillance to attack cancer cells by modulating various components of the immune system, such as cytokines, antigen-presenting cells, and B/T lymphocytes.

Growing evidence indicates that infiltration of effector T lymphocytes is highly correlated with the prognosis of cancer patients [152]. The effector T lymphocyte is a major factor responsible for anti-tumor responses, but essentially faces difficulty in infiltrating into the tumor microenvironment [153]. Staphylococcus endotoxin C2 (SEC2), a super-antigen, activates T lymphocytes as a useful immunotherapeutic agent, that has been traditionally used in China [154]. Song et al. developed the modified recombinant protein ST-4 of SEC2, and applied it with iRGD to enhance the accessibility to the tumor microenvironment [155]. iRGD-mediated ST-4 effectively activated T lymphocytes in mouse B16F10 melanoma cells, and induced lymphocyte infiltration. As a result, the enhanced anticancer effect was achieved through immunosuppression.

Chemotherapeutic agents have been recognized to produce antitumor effects through the direct cytotoxic effect, without eliciting an adaptive or innate immune response [156,157]. However, recent studies emphasized the complex interactions between immune response and cytotoxic agents [158,159]. Anti-tumor immune response in chemotherapeutic treatments is triggered by the death of tumor cells [160,161]. Dendritic cells acquire antigens from apoptotic cancer cells, present it to T cells, and solicit antigen-specific proliferation of T cells. In addition, some chemotherapeutics-induced increase of lymphocyte infiltration in tumor microenvironment can slowly prevent the growth of residual tumor cells and prolong overall survival [162,163]. However, due to non-selective cytotoxicity and non-specific biodistribution, chemotherapeutic agents can cause undesired side effects to the immune system by affecting the spleen and bone marrow [164]. Deng et al. investigated whether co-administration of pirarubicin loaded lipid carriers with iRGD could improve anti-tumor immune response, and reduce the side effects of anti-tumor drugs, and reported an increase in cytotoxic T lymphocyte infiltration and cytokine secretion, which contributed to the extended overall survival of pirarubicin-lipid carrier+iRGD treated mice [165].

Combinatorial treatment with immunomodulators and chemotherapy, i.e., chemoimmunotherapy, showed a synergistic anti-cancer effect, and thus is considered as a promising therapeutic approach for cancer treatment [166,167,168]. As an example, the treatment of co-assembled nanoparticles composed of iRGD derivatives, paclitaxel, and imiquimod resulted in tumor suppression and prevention of recurrence [121]. Here, imiquimod is an immune response modifier inducing the innate immune response. Chemotherapeutic treatment combining an anti-tumor immune response seems to be promising for the complete eradication of metastatic and residual tumors as well as primary tumors [135]. Nano delivery systems in cancer treatment promote innate or adaptive immunity, and inhibit immunosuppression [169,170]. Hu et al. studied a combinatorial approach to treat breast cancer using multistage-responsive nanoparticles. They formulated nanoparticles with doxorubicin, indocyanine green, nitrooxyacetic acid, modified hyaluronic acid, and iRGD. Again, the presence of iRGD achieved the deep penetration of therapeutic agents at the tumor sites, and thus the eradication of primary tumor growth [48].

Immune checkpoints are stimulatory and inhibitory pathways crucial in maintaining the homeostasis of the immune system and self-tolerance. Tumors often evade the immune attack by hijacking inhibitory pathways [171]. In other words, to avoid an immune attack, tumor cells attempt to escape the immune surveillance by editing the tumor microenvironment. Additionally, tumor cells attempt to evade immune recognition; the loss of antigen on tumor cell surfaces allows tumor cells to avoid recognition by cytotoxic T cells [172]. The inhibitory receptor cytotoxic T lymphocyte-associated antigen-4 (CTLA-4) is constitutively expressed on regulatory T cells (Tregs) [173], while it is transiently expressed on activated T cells [174]. CTLA-4 present on exhausted T cells competes with the co-stimulatory receptor CD28. The binding affinity of CTLA-4 is higher than that of CD28. Thus, CTLA-4 binds to the ligands of CD80/CD86 expressed on antigen-presenting cells (APCs), preventing auto-immune responses and leading to T-cell anergy [175,176,177,178]. The overexpression of CTLA-4 on Tregs is highly observed in lung cancer, indicating that CTLA-4 contributes to immune tolerance and immune evasion [179]. Thus, anti-CTLA-4 treatments may restore immune responses to attack tumor cells. Conversely, the inhibitory receptor programmed cell death protein 1 (PD-1) expressed on activated T cells binds to programmed death ligand 1 (PD-L1) and PD-L2 to suppress the activation of cytotoxic T cells [180,181]. Unlike PD-L2, which is primarily expressed on APCs, PD-L1 is expressed on various cells, including tumor cells, which are protected from immune attacks [182,183]. PD-L1 binds to CD80 expressed on T cell surfaces and inhibits functional T cell activation. Thus, the immune evasion signal may be inactivated by blocking the binding events between PD-1 and PD-L1 via the administration of a PD-1 antibody [184]. Immune checkpoint blockades prevent tumor cells from evading the immune attack by activating T cells. Particularly, CTLA-4 and PD-1 inhibitors can stimulate anti-tumor immune responses [185]. Ipilimumab is an immune checkpoint blockade with known anti-CTLA-4 activity. After decades of continuous efforts to translate laboratory findings into clinical practice, FDA approved the use of ipilimumab for advanced melanoma treatment [186]. Since then, there has been an increasing interest in immunotherapy, specifically on PD-1/PDL-1 axis targeting advanced cancer stages [187].

Despite dramatic outcomes by the immunotherapy in some cancer patients, treatments with CTLA-4 or PD-1/PD-L1 immune checkpoint blockers were effective only in about 15% to 25% of patients with various cancers [188]. Tumor heterogeneity, variation in cancer type and stage, and treatment history were suggested to contribute to this unpredictable efficacy of immunotherapy. Substantial efforts were required to amplify the efficacy of immunotherapy in majority of patients with variable cancer types. As an effort to achieve this goal, the investigations focused particularly on the tumor-specific target delivery of immune modulators and the tumor-specific activation of immune systems in the milieu of complex tumor environments. Recent studies have focused on the iRGD peptide as a major player in enhancing the efficacy of immunotherapy. For example, gene delivery therapy against PD-L1 loaded on solid–lipid nanoparticles (SLN) to treat glioblastoma utilized iRGD conjugation and achieved immune activation via the downregulation of PD-L1 [109]. In addition, the co-administration of IL24-iRGD led to significantly increased apoptotic events in non-small cell lung cancer cells compared with the single treatment of IL24 [189]. The enhanced alteration of tumor immune environment, such as the decreases in Treg cells and the increases in CD4 T cells, has been indicated as a major player in this enhanced apoptotic event [190].

However, similar to other chemotherapeutic treatments, the use of immune checkpoint blockades, such as that for CTLA-4, PD-1, and PD-L1, also develops resistance to some extent, because blocking one pathway often activates the others in the complex network of tumor environments. In contrast to the treatment modalities using immune checkpoint blockades, adaptive cell immunotherapy becomes a potential treatment option, which is evident from the positive outcomes and lesser likelihood of causing acquisition of resistance. Nevertheless, adaptive cell transfer requires infiltration of formidable barriers of solid tumors, and has to overcome the immunosuppressive environments. Recently, nanoparticles containing multiple drugs, including the tumor penetrating moiety of iRGD, were suggested to be a practical intervention to create optimal tumor environment for the cancer immunotherapy [191]. In that study, lipid nanoparticles coated with iRGD successfully restored the tumor microenvironments from immunosuppressive to stimulatory, and allowed the tumor-specific chimeric antigen receptor -T (CAR-T) cells to trigger tumor regression and penetrate into the lesion. Ding et al. also reported the synergistic effect of iRGD with PD-1 knockout immunotherapy [192]. They found that iRGD used with PD-1 knockout lymphocytes showed an anti-tumor effect in gastric cancer by promoting tumor-specific lymphocyte infiltration. They proposed that the binding of iRGD with NRP-1 induces tyrosine phosphorylation of vascular endothelial cadherin, which subsequently opens cell contacts and allows lymphocytes to migrate to, and infiltrate the tumor parenchyma. These findings consistently demonstrated that the iRGD peptide effectively enhances anti-tumor efficacy of immunotherapy, thus paving a new avenue in cancer treatment.

### 3.4. iRGD Application in Brain Pathology

Brain tumor remains the biggest challenge in cancer therapy owing to multiple biological obstacles, including the BBB, the blood–brain tumor barrier (BBTB), hypoxia regions, tumor heterogeneity, glioma stem cells, and drug-efflux pumps [193]. Although the BBB acts as a vital filter to control substances that pass through the blood to the brain, it also forms a formidable impediment in drug delivery for the treatment of brain cancer. The BBB is composed of brain capillary endothelial cells, astrocytes, pericytes, and neurons. The endothelial cells are connected by tight junctions and narrow gaps between endothelial cells that make it difficult for macromolecules to pass through [194,195], and block the penetration of hydrophilic molecules (>400 Da) [196]. Meanwhile, the BBTB is more permeable than BBB, because anomalous angiogenesis occurs here due to the upregulated vascular endothelial growth factor, triggered by tumor-induced hypoxia [197]. High-grade glioma shows dysfunctional and heterogeneous BBTB, whereas BBTB of lower-grade gliomas is similar to BBB. Although the BBTB consists of leaky vasculatures, the penetration of therapeutics remains highly limited, thus imposing a challenge on the treatment of gliomas [193,198]. The P-glycoprotein, an efflux pump, is abundantly present in cerebrovascular endothelial cells, and protects the brain from the accumulation of hydrophobic drugs, including chemotherapeutics [199]. The inhibition of drug efflux transporters has been shown to enhance the brain penetration of various drugs [200].

Diverse interventions have been performed to improve drug permeability in the BBB and BBTB. Here, we briefly summarize these representative approaches. Earlier, the osmotic disruption, using the hyperosmotic solution of arabinose and mannitol, was attempted for disrupting the tight junctions of the BBB [201]. However, due to the concerns on transient cerebral edema, and the risk of exacerbating neurological deficits, osmotic disruption is not widely utilized in clinical practices. Alternatively, the physical disruption of the BBB has been attempted through the application of ultrasound or electromagnetic waves [202]. However, similar concerns regarding tumor diffusion and neurotoxic effects were raised. Chemical compounds, such as bradykinin, are known to enhance the BBB permeability by inducing vasodilation [203]. Thus, the inhibition of multidrug efflux transporters was subsequently utilized to enhance the drug penetration through the BBB. Use of transferrin coupled to paclitaxel, a strategy exploiting the receptor-mediated transport system without opening the tight junctions, also elicited endocytosis [196]. In addition, therapeutic agents have been directly injected into the resection cavity of glioma via invasive approach [202]. Nanocarrier-based drug combination strategies, such as liposomes, have also been applied to improve the systemic or local delivery of anti-cancer drugs to cure glioblastoma. The modification of drugs using lipid groups also facilitates brain permeability to some extent [204]. However, this method concomitantly enhances the nonspecific accumulation of drugs in other tissues via systemic circulation [193]. Further, a positive-pressure bulk flow via convection-enhanced delivery (CED) may mediate the local simple diffusion of drugs into brain. However, side effects, such as edema and infection, cannot be avoided by CED [205].

Recently, iRGD has been suggested as a promising candidate to overcome the poor permeability of therapeutics in the brain. Many studies have showed that the iRGD peptide synergistically intensified the BBB disruption through combinations with the abovementioned disruptive interventions, including radiation and ultrasound therapy. As mentioned in Section 3.3, Erel-Akbaba et al. showed that the conjugation of iRGD effectively enhances the uptake of SLNs for the gene delivery of siRNA-PD-L1 to the brain tumor, consequently resulting in a decrease in glioblastoma growth [109]. Radiation primed against glioma also enhanced the uptake of SLN into the brain. The lipophilicity and the positive charge of SLNs exhibited an increase in the cellular uptake of iRGD, and penetration through the BBB. This study revealed that the use of nano-carriers, chemical modification of drugs, radiation, and active targeting using iRGD synergistically improved the penetration of drugs through the BBB, and efficiently optimized the efficacy of immunotherapy. As mentioned earlier, ultrasound waves have been applied to physically disrupt the BBB, and to improve its permeability. The study using the ultrasound-induced sonodynamic therapy (SDT) showed that the iRGD peptide increases the median survival time and chemotherapeutic efficacy of SDT in the treatment of gliomas [153]. SDT with iRGD-modified liposomes has also been shown to effectively promote the penetration of drugs into glioma cells. Together with the combination of radiation therapy and the iRGD peptide, this result supports that the combined therapy with the iRGD peptide and physical disruption of BBB synergistically contributed to the enhanced permeability of the BBB and the efficacy of chemotherapeutics.

Several studies have shown that the iRGD peptide has a potential to advance cancer diagnosis and prognosis because it has enhanced the imaging resolution in magnetic resonance imaging (MRI) [206,207]. MRI is a powerful tool that visualizes cellular density, but has a limitation in sensitivity and resolution. iRGD can trace invasive lesions in tumor because it binds to integrins overexpressed on newly generated tumor blood vessels. Nanoparticles coated with the iRGD peptide have been used to increase the resolution of MRI, thus revealing metastatic lesions of the brain from breast cancer [206]. In addition, a study has showed that intravenously administered iRGD-nanoparticles effectively suppress tumor growth at the initial stages of metastasis. These results stated that the iRGD peptide is beneficial not only for imaging enhancement, but also for preventive alleviation of metastatic progression of breast cancer. Moncelet et al. revealed that the internalization of iRGD-Alexa488 into U87 cells highly correlates with cell density, which can be used as a parameter to determine the grade and invasiveness of glioma [207]. They reported that the iRGD peptide significantly improved the signal-to-noise ratio of MRI contrast agents, such as gadolinium chelates.

Numerous studies have reported that the active targeting using the iRGD peptide yields enhanced therapeutic outcomes in brain cancer treatment owing to increased cell internalization and BBB penetration. For example, the iRGD-linked polymeric micelles loaded with platinum-based chemotherapeutics were found to be actively internalized, and thus exhibited high anti-tumor effect in an orthotopic mouse glioblastoma model, compared with polymeric micelles without iRGD [103]. In addition, the iRGD-conjugated prodrug micelles encapsulating camptothecin effectively penetrated the BBB to arrive at glioma sites [104]. These results consistently indicated that the iRGD-conjugated micelles effectively promoted the transport of the drugs through the BBB, thus suggesting a promising approach for glioma therapy. Another study examined the anti-glioma effects of conjugated iRGD with lycobetaine and octreotide liposomes (LBT-OCT-LPs-nRGD) [116], and showed that the LBT-OCT-LPs-nRGD significantly enhanced the survival time, and reduced tumor-associated macrophages.

## 4. Conclusions and Future Perspectives

The tumor microenvironment is a complex milieu composed of cellular and acellular components with unique structure and physiology, which provides obstacles in the delivery of chemotherapeutic agents. As an emerging technology, the iRGD peptide, containing the integrin-binding RGD and the cryptic CendR motif, is a novel strategy that overcomes the poor penetration of chemotherapeutics into the tumor parenchyma. The integrins highly expressed on tumor endothelium cells enable iRGD to home in on and access the tumor site. Moreover, the overexpressed NRP-1 receptors on various types of cancer cells can mediate endocytosis and lead to deep penetration into tumor tissues. Therefore, the iRGD-mediated active delivery depends not only on the overexpressed integrins at the tumor angiogenetic blood vessels, but also on the NRP-1-mediated endocytosis at tumor sites. This dual-targeting function makes iRGD superior to the conventional RGD, which only targets integrins. Nevertheless, the tumor targeting capability of iRGD highly depends on the level of NRP-1 expression, which varies greatly depending on the type and stage of the cancer [84]. Individual differences in its expression exacerbate the simple prediction of treatment outcomes. Thus, in order to clinically utilize iRGD in a wide range of cancer treatments, a systematic evaluation of NRP-1 expression according to cancer type and progression should be performed. In addition, the concept of personalized medicine would be beneficial for the use of iRGD through preemptive investigation of its expression in individual patients.

The application of the iRGD peptide can advance the treatment modalities and the targeting activity of engineered delivery vehicles through covalent conjugation, or co-administration with chemotherapeutic agents and delivery vehicles. Thus far, accumulating evidences seem to support that these two approaches equally enhance tumor penetration and efficacy of chemotherapeutic agents. A study suggested that conjugation might add complexity to surface of nanocarries that might result in the unexpected effect in the complicated in vivo system [208]. Moreover, the transportation of conjugated drugs may be affected by the relatively limited number of target receptors on the vasculature, while separate injection of the free peptide can trigger bulk transportation of therapeutic drugs at the tumor site [54,209]. However, only a few studies compared the co-administration and conjugation approaches directly. Thoroughly designed studies are required to reveal the specific mechanisms by which the co-administered and the conjugated iRGD contribute to the deep penetration of drugs into tumor sites.

A recent pioneering approach using iRGD has been applied in immunotherapy and gene delivery therapy. Despite dramatic outcomes following cancer immunotherapy in some patients, its clinical success has been lower than expected because of the complexity of the tumor microenvironment. T lymphocyte infiltration through the tumor parenchyma plays a critical role in the anticancer effects of immunotherapeutic agents. Interestingly, co-administration of iRGD enhanced T lymphocyte infiltration by enabling intercellular contact of cadherin tyrosine phosphorylation. Moreover, iRGD conjugation induces immune activation by downregulating PD-L1. Nevertheless, it is critical to investigate the underlying mechanism of iRGD in the immune response to clarify the synergistic role of iRGD in immunotherapy.

In contrast, iRGD-mediated transport shows potential for resolving long-standing limitations to drug delivery to the brain. In fact, some interventions to improve drug permeability in the BBB and BBTB including hyperosmotic solution, electromagnetic waves, and bradykinin affect BBB permeability over a wide spectrum. Unfortunately, these interventions led to undesired problems such as nonspecific permeability, simple diffusion, and side effects. In contrast, the iRGD peptide enhanced the BBB permeability for specific target drugs. The combinatorial regimen with other BBB disruptive methods, including radioactive and ultrasonic therapy, synergistically enhanced the penetration of chemotherapeutics through the BBB. Additionally, combining iRGD peptides with nanoparticle-based delivery vehicles or lipophilic modification of drugs can augment the penetration of drugs into the brain.

Phase 1 clinical trial is currently evaluating the efficacy and safety of iRGD for PDAC patients. The study is mainly monitoring the dose escalation of iRGD for combinatorial treatment with gemcitabine and nab-paclitaxel. Considering that interpersonal variations in NRP-1 expression highly affect the success of clinical trials, the expression of NRP-1 at each patient is monitored by immunohistochemistry of tumor biopsy. The successful completion of this clinical trial will determine the clinical fate of iRGD peptide for the treatment of various cancers. In conclusion, the combination of iRGD dual-targeting technique with various chemotherapeutics and/or new treatment modalities is expected to open a new era in the fight against cancer.

## Figures and Tables

**Table 1:** Application of iRGD conjugation with therapeutic agents to improve the drug efficacy.

Vehicles	API^a^	Cancer Type	Ref
Liposomes	PEDF^b^ DNA	Colorectal cancer (CT26)	[97]
	none	Breast cancer (4T1)	[98]
	Doxorubicin	Breast cancer (4T1), melanoma (B16-F10)	[107,113,114,115]
	Lycobetaine Octreotide	Glioma (C6)	[116]
Polymeric NPs^c^	Doxorubicin	Breast cancer (4T1), liver cancer (VX2)	[112,117,118,119]
	Paclitaxel	Gastric cancer (MKN-45P), colon cancer (CT26), hepatoma (H22), breast cancer (4T1)	[110,120,121]
	Carmustine O6-enzylguanine	Glioma (F98, C6, U87)	[122]
	Tamoxifen	Breast cancer (MCF-7, T47D)	[123]
	Isoliquiritigenin	Breast cancer (4T1)	[111]
Silica NPs^c^	Doxorubicin	Colorectal cancer (HT-29)	[124]
Micelles	Paclitaxel	Prostate cancer (PC-3, PPC1), pancreatic cancer (MIA PaCa-2), breast cancer (BT474)	[52]
	Platinum complex	Glioblastoma (U87)	[103]
	Camptothecin	Glioblastoma (U87)	[104]
	Docetaxel	HeLa	[108]
Hydrogels	Doxorubicin	Melanoma (B16)	[125]
	Gambogic acid	Gastric cancer (MKN-45)	[126]
Solid lipid NPs^c^	siRNA	Glioblastoma (GL261)	[109]
Protein NCs^d^	Paclitaxel	Hepatoma (H22)	[127]

API^a^, Active pharmaceutical ingredient; PEDF^b^, Pigment epithelium-derived factor; NPs^c^, Nanoparticles; NCs^d^, Nanocapsules.

**Table 2:** Application of iRGD co-administration with therapeutic agents to improve the drug efficacy.

Drug	Vehicles	API^a^	Cancer Type	Ref
drug delivery systems	Polymeric NPs^b^	Paclitaxel	Glioma (C6), breast cancer (BT474), colorectal cancer (LS174T)	[128,129]
		Doxorubicin	Breast cancer (4T1)	[130]
	Silica NPs^b^	Irinotecan	Pancreatic KPC-derived cancer	[131]
	Liposomes	Doxorubicin	Prostate cancer (22Rv1), melanoma (B16F10)	[54,132]
	Polypeptide NPs^b^	Cisplatin	Melanoma (B16F1)	[133]
	Gold NPs^b^ (Au NPs)	Doxorubicin	Breast cancer (4T1)	[134]
	Dendrimers	Doxorubicin	Prostate cancer (22Rv1), Melanoma (B16F10)	[135]
Drugs only	None	HPRP-A1^c^	Non-small cell lung cancer (A549)	[48]
		Gemcitabine	Non-small cell lung cancer (A549)	[136]
		Cetuximab	Non-small cell lung cancer (A549)	[137]
		izTRAIL^d^ & Sorafenib	Fibrosarcoma (HT-1080)	[138]

API^a^, Active pharmaceutical ingredient; NPs^b^, Nanoparticles; HPRP-A1^c^, anti-microbial peptide derived from the N-terminus of ribosomal protein L1 of *Helicobacter pylori*; TRAIL^d^, TNF-α-related apoptosis inducing ligand.

## Abbreviations

The following abbreviations are used in this manuscript:

APCs	Antigen-presenting cells
API	Active pharmaceutical ingredient
BBB	Blood–brain barrier
CendR	C-end Rule
CTLA-4	Cytotoxic T lymphocyte-associated antigen-4
DSG2	Desmoglein 2
ECM	Extracellular matrix
EPR	Enhanced permeability and retention
FAM	Fluorescein
GF	Gland formation
nGF	No gland formation
HA-PLA	Self-assembled amphiphilic block copolymer NPs
HPRP-A1	Anti-microbial peptide derived from the N-terminus of ribosomal protein L1 of *H. pylori*
IFP	Interstitial fluid pressure
iRGD-ICG-LPs	iRGD-modified indocyanine green (ICG) liposomes
ISL-iRGD NPs	iRGD-modified lipid–polymer hybrid NPs loaded with isoliquiritigenin
JO-1	Junction opener-1
LGR	Leucine-rich repeat-containing G protein receptors
NCs	Nanocapsules
NIR	Near infrared
NPs	Nanoparticles
NRP-1	Neuropilin-1
PCL-PVP	Poly (ε-caprolactone)-poly (N-vinylpyrrolidone)
PD-1	Programmed cell death protein 1
PD-L1	Programmed death ligand 1
PDAC	Pancreatic ductal adenocarcinoma
PDT	Photodynamic therapy
PEDF	Pigment epithelium-derived factor
PLGA	Paclitaxel-loaded poly (lactic-co-glycolic acid)
PTT	Photothermal therapy
R-LP	iRGD-modified liposomes
R-LP/PEDF	iRGD-conjugated pigment epithelium-derived factor (PEDF)-DNA-loaded liposomes
R-LP/shelF3i	iRGD-modified liposomes encapsulating elF3i shRNA
SEC2	*Staphylococcus* endotoxin C2
SEER	Surveillance, Epidemiology, and End Results
SLN	Solid–lipid nanoparticles
TAMs	Tumor-associated macrophages
TRAIL	TNF-α-related apoptosis inducing ligand
VEGF	Vascular endothelial growth factor

## References

[1] Jung K.W., Won Y.J., Kong H.J., Lee E.S. (2019). Cancer Statistics in Korea: Incidence, Mortality, Survival, and Prevalence in 2016. Cancer Res. Treat..

[2] White M.C., Holman D.M., Boehm J.E., Peipins L.A., Grossman M., Henley S.J. (2014). Age and cancer risk: A potentially modifiable relationship. Am. J. Prev. Med..

[3] Zhang H.-T., Li H.-C., Li Z.-W., Guo C.-H. (2011). A tumor-penetrating peptide modification enhances the antitumor activity of endostatin in vivo. Anticancer Drugs.

[4] Rabbani-Chadegani A., Paydar P., Amirshenava M., Aramvash A. (2015). An in vitro study on the effect of vinca alkaloid, vinorelbine, on chromatin histone, HMGB proteins and induction of apoptosis in mice non-adherent bone marrow cells. Drug Chem. Toxicol..

[5] Nyongesa C.O., Park S. (2018). Chemotherapeutic resistance: A nano-mechanical point of view. Biol. Chem..

[6] Hambley T.W., Hait W.N. (2009). Is anticancer drug development heading in the right direction?. Cancer Res..

[7] Bissell M.J., Radisky D. (2001). Putting tumours in context. Nat. Rev. Cancer.

[8] Mueller M.M., Fusenig N.E. (2004). Friends or foes—Bipolar effects of the tumour stroma in cancer. Nat. Rev. Cancer.

[9] Madara J.L. (1998). Regulation of the movement of solutes across tight junctions. Annu. Rev. Physiol..

[10] Harada H., Iwatsuki K., Ohtsuka M., Han G.W., Kaneko F. (1996). Abnormal desmoglein expression by squamous cell carcinoma cells. Acta Derm. Venereol..

[11] Biedermann K., Vogelsang H., Becker I., Plaschke S., Siewert J.R., Hofler H., Keller G. (2005). Desmoglein 2 is expressed abnormally rather than mutated in familial and sporadic gastric cancer. J. Pathol..

[12] Pluen A., Boucher Y., Ramanujan S., McKee T.D., Gohongi T., di Tomaso E., Brown E.B., Izumi Y., Campbell R.B., Berk D.A. (2001). Role of tumor-host interactions in interstitial diffusion of macromolecules: Cranial vs. subcutaneous tumors. Proc. Natl. Acad. Sci. USA.

[13] Jain R.K. (1990). Vascular and interstitial barriers to delivery of therapeutic agents in tumors. Cancer Metastasis Rev..

[14] Wenning L.A., Murphy R.M. (1999). Coupled cellular trafficking and diffusional limitations in delivery of immunotoxins to multicell tumor spheroids. Biotechnol. Bioeng..

[15] Kuh H.J., Jang S.H., Wientjes M.G., Weaver J.R., Au J.L. (1999). Determinants of paclitaxel penetration and accumulation in human solid tumor. J. Pharmacol. Exp. Ther..

[16] Huang S., Ingber D.E. (2005). Cell tension, matrix mechanics, and cancer development. Cancer Cell.

[17] Levental K.R., Yu H., Kass L., Lakins J.N., Egeblad M., Erler J.T., Fong S.F., Csiszar K., Giaccia A., Weninger W. (2009). Matrix crosslinking forces tumor progression by enhancing integrin signaling. Cell.

[18] Lopez J.I., Kang I., You W.K., McDonald D.M., Weaver V.M. (2011). In situ force mapping of mammary gland transformation. Integr. Biol..

[19] Netti P.A., Berk D.A., Swartz M.A., Grodzinsky A.J., Jain R.K. (2000). Role of extracellular matrix assembly in interstitial transport in solid tumors. Cancer Res..

[20] Brown E.B., Boucher Y., Nasser S., Jain R.K. (2004). Measurement of macromolecular diffusion coefficients in human tumors. Microvasc. Res..

[21] Heldin C.H., Rubin K., Pietras K., Ostman A. (2004). High interstitial fluid pressure—An obstacle in cancer therapy. Nat. Rev. Cancer.

[22] Matsumura Y., Maeda H. (1986). A new concept for macromolecular therapeutics in cancer chemotherapy: Mechanism of tumoritropic accumulation of proteins and the antitumor agent smancs. Cancer Res..

[23] Maeda H., Sawa T., Konno T. (2001). Mechanism of tumor-targeted delivery of macromolecular drugs, including the EPR effect in solid tumor and clinical overview of the prototype polymeric drug SMANCS. J. Control. Release.

[24] Maeda H., Bharate G.Y., Daruwalla J. (2009). Polymeric drugs for efficient tumor-targeted drug delivery based on EPR-effect. Eur. J. Pharm. Biopharm..

[25] Torchilin V.P. (2000). Drug targeting. Eur. J. Pharm. Sci..

[26] Bae Y.H. (2009). Drug targeting and tumor heterogeneity. J. Control. Release.

[27] Gabizon A., Catane R., Uziely B., Kaufman B., Safra T., Cohen R., Martin F., Huang A., Barenholz Y. (1994). Prolonged circulation time and enhanced accumulation in malignant exudates of doxorubicin encapsulated in polyethylene-glycol coated liposomes. Cancer Res..

[28] Von Hoff D.D., Ervin T., Arena F.P., Chiorean E.G., Infante J., Moore M., Seay T., Tjulandin S.A., Ma W.W., Saleh M.N. (2013). Increased survival in pancreatic cancer with nab-paclitaxel plus gemcitabine. N. Engl. J. Med..

[29] Wang-Gillam A., Li C.P., Bodoky G., Dean A., Shan Y.S., Jameson G., Macarulla T., Lee K.H., Cunningham D., Blanc J.F. (2016). Nanoliposomal irinotecan with fluorouracil and folinic acid in metastatic pancreatic cancer after previous gemcitabine-based therapy (NAPOLI-1): A global, randomised, open-label, phase 3 trial. Lancet.

[30] Adams G.P., Schier R., McCall A.M., Simmons H.H., Horak E.M., Alpaugh R.K., Marks J.D., Weiner L.M. (2001). High affinity restricts the localization and tumor penetration of single-chain fv antibody molecules. Cancer Res..

[31] Gosk S., Moos T., Gottstein C., Bendas G. (2008). VCAM-1 directed immunoliposomes selectively target tumor vasculature in vivo. Biochim. Biophys. Acta.

[32] Lammers T., Hennink W.E., Storm G. (2008). Tumour-targeted nanomedicines: Principles and practice. Br. J. Cancer.

[33] Danhier F., Feron O., Preat V. (2010). To exploit the tumor microenvironment: Passive and active tumor targeting of nanocarriers for anti-cancer drug delivery. J. Control. Release.

[34] Bae Y.H., Park K. (2011). Targeted drug delivery to tumors: Myths, reality and possibility. J. Control. Release.

[35] Choi I.K., Strauss R., Richter M., Yun C.O., Lieber A. (2013). Strategies to increase drug penetration in solid tumors. Front. Oncol..

[36] Van Der Westhuizen E.T., Summers R.J., Halls M.L., Bathgate R.A., Sexton P.M. (2007). Relaxin receptors—New drug targets for multiple disease states. Curr. Drug Targets.

[37] Amento E.P., Bhan A.K., McCullagh K.G., Krane S.M. (1985). Influences of gamma interferon on synovial fibroblast-like cells. Ia induction and inhibition of collagen synthesis. J. Clin. Investig..

[38] Kim J.H., Lee Y.S., Kim H., Huang J.H., Yoon A.R., Yun C.O. (2006). Relaxin expression from tumor-targeting adenoviruses and its intratumoral spread, apoptosis induction, and efficacy. J. Natl. Cancer Inst..

[39] Silvertown J.D., Ng J., Sato T., Summerlee A.J., Medin J.A. (2006). H2 relaxin overexpression increases in vivo prostate xenograft tumor growth and angiogenesis. Int. J. Cancer.

[40] Binder C., Hagemann T., Husen B., Schulz M., Einspanier A. (2002). Relaxin enhances in-vitro invasiveness of breast cancer cell lines by up-regulation of matrix metalloproteases. Mol. Hum. Reprod.

[41] Wang H., Li Z., Yumul R., Lara S., Hemminki A., Fender P., Lieber A. (2011). Multimerization of adenovirus serotype 3 fiber knob domains is required for efficient binding of virus to desmoglein 2 and subsequent opening of epithelial junctions. J. Virol..

[42] Beyer I., van Rensburg R., Strauss R., Li Z., Wang H., Persson J., Yumul R., Feng Q., Song H., Bartek J. (2011). Epithelial junction opener JO-1 improves monoclonal antibody therapy of cancer. Cancer Res..

[43] Wang H., Li Z.Y., Liu Y., Persson J., Beyer I., Moller T., Koyuncu D., Drescher M.R., Strauss R., Zhang X.B. (2011). Desmoglein 2 is a receptor for adenovirus serotypes 3, 7, 11 and 14. Nat. Med..

[44] Beyer I., Cao H., Persson J., Song H., Richter M., Feng Q., Yumul R., van Rensburg R., Li Z., Berenson R. (2012). Coadministration of epithelial junction opener JO-1 improves the efficacy and safety of chemotherapeutic drugs. Clin. Cancer Res..

[45] Li Z., Di C., Li S., Yang X., Nie G. (2019). Smart Nanotherapeutic Targeting of Tumor Vasculature. Acc. Chem. Res..

[46] Zuo H.D., Yao W.W., Chen T.W., Zhu J., Zhang J.J., Pu Y., Liu G., Zhang X.M. (2014). The effect of superparamagnetic iron oxide with iRGD peptide on the labeling of pancreatic cancer cells in vitro: A preliminary study. BioMed Res. Int..

[47] Yin H., Yang J., Zhang Q., Yang J., Wang H., Xu J., Zheng J. (2017). iRGD as a tumorpenetrating peptide for cancer therapy (Review). Mol. Med. Rep..

[48] Hu C., Chen X., Huang Y., Chen Y. (2018). Co-administration of iRGD with peptide HPRP-A1 to improve anticancer activity and membrane penetrability. Sci. Rep..

[49] Sugahara K.N., Scodeller P., Braun G.B., De Mendoza T.H., Yamazaki C.M., Kluger M.D., Kitayama J., Alvarez E., Howell S.B., Teesalu T. (2015). A tumor-penetrating peptide enhances circulation-independent targeting of peritoneal carcinomatosis. J. Control. Release.

[50] Arap W., Pasqualini R., Ruoslahti E. (1998). Cancer treatment by targeted drug delivery to tumor vasculature in a mouse model. Science.

[51] Sipkins D.A., Cheresh D.A., Kazemi M.R., Nevin L.M., Bednarski M.D., Li K.C. (1998). Detection of tumor angiogenesis in vivo by alphaVbeta3-targeted magnetic resonance imaging. Nat. Med..

[52] Sugahara K.N., Teesalu T., Karmali P.P., Kotamraju V.R., Agemy L., Girard O.M., Hanahan D., Mattrey R.F., Ruoslahti E. (2009). Tissue-penetrating delivery of compounds and nanoparticles into tumors. Cancer Cell.

[53] Hoffman J.A., Giraudo E., Singh M., Zhang L., Inoue M., Porkka K., Hanahan D., Ruoslahti E. (2003). Progressive vascular changes in a transgenic mouse model of squamous cell carcinoma. Cancer Cell.

[54] Sugahara K.N., Teesalu T., Karmali P.P., Kotamraju V.R., Agemy L., Greenwald D.R., Ruoslahti E. (2010). Coadministration of a tumor-penetrating peptide enhances the efficacy of cancer drugs. Science.

[55] Teesalu T., Sugahara K.N., Kotamraju V.R., Ruoslahti E. (2009). C-end rule peptides mediate neuropilin-1-dependent cell, vascular, and tissue penetration. Proc. Natl. Acad. Sci. USA.

[56] Calderwood D.A. (2004). Integrin activation. J. Cell Sci..

[57] Nieberler M., Reuning U., Reichart F., Notni J., Wester H.J., Schwaiger M., Weinmuller M., Rader A., Steiger K., Kessler H. (2017). Exploring the Role of RGD-Recognizing Integrins in Cancer. Cancers.

[58] Huveneers S., Danen E.H. (2009). Adhesion signaling—Crosstalk between integrins, Src and Rho. J. Cell Sci..

[59] Geiger B., Spatz J.P., Bershadsky A.D. (2009). Environmental sensing through focal adhesions. Nat. Rev. Mol. Cell Biol..

[60] Schittenhelm J., Klein A., Tatagiba M.S., Meyermann R., Fend F., Goodman S.L., Sipos B. (2013). Comparing the expression of integrins alphavbeta3, alphavbeta5, alphavbeta6, alphavbeta8, fibronectin and fibrinogen in human brain metastases and their corresponding primary tumors. Int. J. Clin. Exp. Pathol..

[61] Pytela R., Pierschbacher M.D., Ruoslahti E. (1985). Identification and isolation of a 140 kd cell surface glycoprotein with properties expected of a fibronectin receptor. Cell.

[62] Boger C., Warneke V.S., Behrens H.M., Kalthoff H., Goodman S.L., Becker T., Rocken C. (2015). Integrins alphavbeta3 and alphavbeta5 as prognostic, diagnostic, and therapeutic targets in gastric cancer. Gastric Cancer.

[63] Schnell O., Krebs B., Wagner E., Romagna A., Beer A.J., Grau S.J., Thon N., Goetz C., Kretzschmar H.A., Tonn J.C. (2008). Expression of integrin alphavbeta3 in gliomas correlates with tumor grade and is not restricted to tumor vasculature. Brain Pathol..

[64] Boger C., Kalthoff H., Goodman S.L., Behrens H.M., Rocken C. (2014). Integrins and their ligands are expressed in non-small cell lung cancer but not correlated with parameters of disease progression. Virchows Arch..

[65] Hosotani R., Kawaguchi M., Masui T., Koshiba T., Ida J., Fujimoto K., Wada M., Doi R., Imamura M. (2002). Expression of integrin alphaVbeta3 in pancreatic carcinoma: Relation to MMP-2 activation and lymph node metastasis. Pancreas.

[66] Hess K., Boger C., Behrens H.M., Rocken C. (2014). Correlation between the expression of integrins in prostate cancer and clinical outcome in 1284 patients. Ann. Diagn Pathol..

[67] Berghoff A.S., Kovanda A.K., Melchardt T., Bartsch R., Hainfellner J.A., Sipos B., Schittenhelm J., Zielinski C.C., Widhalm G., Dieckmann K. (2014). Alphavbeta3, alphavbeta5 and alphavbeta6 integrins in brain metastases of lung cancer. Clin. Exp. Metastasis.

[68] Xiong J.P., Stehle T., Zhang R., Joachimiak A., Frech M., Goodman S.L., Arnaout M.A. (2002). Crystal structure of the extracellular segment of integrin alpha Vbeta3 in complex with an Arg-Gly-Asp ligand. Science.

[69] Vander Kooi C.W., Jusino M.A., Perman B., Neau D.B., Bellamy H.D., Leahy D.J. (2007). Structural basis for ligand and heparin binding to neuropilin B domains. Proc. Natl. Acad. Sci. USA.

[70] Haspel N., Zanuy D., Nussinov R., Teesalu T., Ruoslahti E., Aleman C. (2011). Binding of a C-end rule peptide to the neuropilin-1 receptor: A molecular modeling approach. Biochemistry.

[71] Koivunen E., Gay D.A., Ruoslahti E. (1993). Selection of peptides binding to the alpha 5 beta 1 integrin from phage display library. J. Biol. Chem..

[72] Koivunen E., Wang B., Ruoslahti E. (1995). Phage libraries displaying cyclic peptides with different ring sizes: Ligand specificities of the RGD-directed integrins. Biotechnology.

[73] Salikhova A., Wang L., Lanahan A.A., Liu M., Simons M., Leenders W.P., Mukhopadhyay D., Horowitz A. (2008). Vascular endothelial growth factor and semaphorin induce neuropilin-1 endocytosis via separate pathways. Circ. Res..

[74] Feron O. (2010). Tumor-penetrating peptides: A shift from magic bullets to magic guns. Sci. Transl. Med..

[75] Takagi S., Hirata T., Agata K., Mochii M., Eguchi G., Fujisawa H. (1991). The A5 antigen, a candidate for the neuronal recognition molecule, has homologies to complement components and coagulation factors. Neuron.

[76] Neufeld G., Kessler O. (2008). The semaphorins: Versatile regulators of tumour progression and tumour angiogenesis. Nat. Rev. Cancer.

[77] Gaur P., Bielenberg D.R., Samuel S., Bose D., Zhou Y., Gray M.J., Dallas N.A., Fan F., Xia L., Lu J. (2009). Role of class 3 semaphorins and their receptors in tumor growth and angiogenesis. Clin. Cancer Res..

[78] Kitsukawa T., Shimono A., Kawakami A., Kondoh H., Fujisawa H. (1995). Overexpression of a membrane protein, neuropilin, in chimeric mice causes anomalies in the cardiovascular system, nervous system and limbs. Development.

[79] Kawasaki T., Kitsukawa T., Bekku Y., Matsuda Y., Sanbo M., Yagi T., Fujisawa H. (1999). A requirement for neuropilin-1 in embryonic vessel formation. Development.

[80] Soker S., Takashima S., Miao H.Q., Neufeld G., Klagsbrun M. (1998). Neuropilin-1 is expressed by endothelial and tumor cells as an isoform-specific receptor for vascular endothelial growth factor. Cell.

[81] Poon R.T., Fan S.T., Wong J. (2001). Clinical implications of circulating angiogenic factors in cancer patients. J. Clin. Oncol..

[82] Bachelder R.E., Crago A., Chung J., Wendt M.A., Shaw L.M., Robinson G., Mercurio A.M. (2001). Vascular endothelial growth factor is an autocrine survival factor for neuropilin-expressing breast carcinoma cells. Cancer Res..

[83] Zhang H., He C., Han S., Zhu M., Ke W. (2016). The prognostic value of neuropilin-1 in various cancers: A meta-analysis. Int. J. Clin. Exp. Med..

[84] Akashi Y., Oda T., Ohara Y., Miyamoto R., Kurokawa T., Hashimoto S., Enomoto T., Yamada K., Satake M., Ohkohchi N. (2014). Anticancer effects of gemcitabine are enhanced by co-administered iRGD peptide in murine pancreatic cancer models that overexpressed neuropilin-1. Br. J. Cancer.

[85] Seo H.S., Hyeon J., Song I.H., Lee H.H. (2020). Relationship between neuropilin-1 expression and prognosis, according to gastric cancer histology. J. Mol. Histol..

[86] Hanahan D., Folkman J. (1996). Patterns and emerging mechanisms of the angiogenic switch during tumorigenesis. Cell.

[87] Raghunand N., Gatenby R.A., Gillies R.J. (2003). Microenvironmental and cellular consequences of altered blood flow in tumours. Br. J. Radiol..

[88] Harris A.L. (2002). Hypoxia—A key regulatory factor in tumour growth. Nat. Rev. Cancer.

[89] Carmeliet P. (2000). Mechanisms of angiogenesis and arteriogenesis. Nat. Med..

[90] Desgrosellier J.S., Cheresh D.A. (2010). Integrins in cancer: Biological implications and therapeutic opportunities. Nat. Rev. Cancer.

[91] Avraamides C.J., Garmy-Susini B., Varner J.A. (2008). Integrins in angiogenesis and lymphangiogenesis. Nat. Rev. Cancer.

[92] Stollman T.H., Ruers T.J., Oyen W.J., Boerman O.C. (2009). New targeted probes for radioimaging of angiogenesis. Methods.

[93] Wang K., Zhang X., Liu Y., Liu C., Jiang B., Jiang Y. (2014). Tumor penetrability and anti-angiogenesis using iRGD-mediated delivery of doxorubicin-polymer conjugates. Biomaterials.

[94] Laakkonen P., Porkka K., Hoffman J.A., Ruoslahti E. (2002). A tumor-homing peptide with a targeting specificity related to lymphatic vessels. Nat. Med..

[95] Alberici L., Roth L., Sugahara K.N., Agemy L., Kotamraju V.R., Teesalu T., Bordignon C., Traversari C., Rizzardi G.P., Ruoslahti E. (2013). De novo design of a tumor-penetrating peptide. Cancer Res..

[96] Xiao W., Zhang W., Huang H., Xie Y., Zhang Y., Guo X., Jin C., Liao X., Yao S., Chen G. (2020). Cancer Targeted Gene Therapy for Inhibition of Melanoma Lung Metastasis with eIF3i shRNA Loaded Liposomes. Mol. Pharm..

[97] Bao X., Zeng J., Huang H., Ma C., Wang L., Wang F., Liao X., Song X. (2020). Cancer-targeted PEDF-DNA therapy for metastatic colorectal cancer. Int. J. Pharm..

[98] Yan F., Wu H., Liu H., Deng Z., Liu H., Duan W., Liu X., Zheng H. (2016). Molecular imaging-guided photothermal/photodynamic therapy against tumor by iRGD-modified indocyanine green nanoparticles. J. Control. Release.

[99] Shen J., Meng Q., Sui H., Yin Q., Zhang Z., Yu H., Li Y. (2014). iRGD conjugated TPGS mediates codelivery of paclitaxel and survivin shRNA for the reversal of lung cancer resistance. Mol. Pharm..

[100] Wang C.-F., Sarparanta M.P., Mäkilä E.M., Hyvönen M.L., Laakkonen P.M., Salonen J.J., Hirvonen J.T., Airaksinen A.J., Santos H.A. (2015). Multifunctional porous silicon nanoparticles for cancer theranostics. Biomaterials.

[101] Li X., Wu M., Pan L., Shi J. (2016). Tumor vascular-targeted co-delivery of anti-angiogenesis and chemotherapeutic agents by mesoporous silica nanoparticle-based drug delivery system for synergetic therapy of tumor. Int. J. Nanomed..

[102] Liu X., Jiang J., Ji Y., Lu J., Chan R., Meng H. (2017). Targeted drug delivery using iRGD peptide for solid cancer treatment. Mol. Syst. Des. Eng..

[103] Miura Y., Takenaka T., Toh K., Wu S., Nishihara H., Kano M.R., Ino Y., Nomoto T., Matsumoto Y., Koyama H. (2013). Cyclic RGD-linked polymeric micelles for targeted delivery of platinum anticancer drugs to glioblastoma through the blood-brain tumor barrier. ACS Nano.

[104] Lu L., Zhao X., Fu T., Li K., He Y., Luo Z., Dai L., Zeng R., Cai K. (2020). An iRGD-conjugated prodrug micelle with blood-brain-barrier penetrability for anti-glioma therapy. Biomaterials.

[105] Irby D., Du C., Li F. (2017). Lipid-Drug Conjugate for Enhancing Drug Delivery. Mol. Pharm..

[106] Han S.-M., Na Y.-G., Lee H.-S., Son G.-H., Jeon S.-H., Bang K.-H., Kim S.-J., Lee H.-J., Cho C.-W. (2018). Improvement of cellular uptake of hydrophilic molecule, calcein, formulated by liposome. J. Pharm. Investig..

[107] Song X., Wan Z., Chen T., Fu Y., Jiang K., Yi X., Ke H., Dong J., Yang L., Li L. (2016). Development of a multi-target peptide for potentiating chemotherapy by modulating tumor microenvironment. Biomaterials.

[108] Wang L., Xie X., Liu D., Fang X.-B., Li P., Wan J.-B., He C.-W., Chen M.-W. (2016). iRGD-mediated reduction-responsive DSPE–PEG/LA–PLGA–TPGS mixed micelles used in the targeted delivery and triggered release of docetaxel in cancer. RSC Adv..

[109] Liu Y., Ji M., Wong M.K., Joo K.I., Wang P. (2013). Enhanced therapeutic efficacy of iRGD-conjugated crosslinked multilayer liposomes for drug delivery. BioMed Res. Int..

[110] Yu K.F., Zhang W.Q., Luo L.M., Song P., Li D., Du R., Ren W., Huang D., Lu W.L., Zhang X. (2013). The antitumor activity of a doxorubicin loaded, iRGD-modified sterically-stabilized liposome on B16-F10 melanoma cells: In vitro and in vivo evaluation. Int. J. Nanomed..

[111] Dai W., Fan Y., Zhang H., Wang X., Zhang Q., Wang X. (2015). A comprehensive study of iRGD-modified liposomes with improved chemotherapeutic efficacy on B16 melanoma. Drug Deliv..

[112] Chen T., Song X., Gong T., Fu Y., Yang L., Zhang Z., Gong T. (2017). nRGD modified lycobetaine and octreotide combination delivery system to overcome multiple barriers and enhance anti-glioma efficacy. Colloids Surf. B Biointerfaces.

[113] Nie X., Zhang J., Xu Q., Liu X., Li Y., Wu Y., Chen C. (2014). Targeting peptide iRGD-conjugated amphiphilic chitosan-co-PLA/DPPE drug delivery system for enhanced tumor therapy. J. Mater. Chem. B.

[114] Zhang T., Lip H., He C., Cai P., Wang Z., Henderson J.T., Rauth A.M., Wu X.Y. (2019). Multitargeted Nanoparticles Deliver Synergistic Drugs across the Blood-Brain Barrier to Brain Metastases of Triple Negative Breast Cancer Cells and Tumor-Associated Macrophages. Adv. Health Mater..

[115] Chen J., Yang Q., Liu M., Lin M., Wang T., Zhang Z., Zhong X., Guo N., Lu Y., Xu J. (2019). Remarkable Boron Delivery Of iRGD-Modified Polymeric Nanoparticles for Boron Neutron Capture Therapy. Int. J. Nanomed..

[116] Xie Y., Qi X., Xu K., Meng X., Chen X., Wang F., Zhong H. (2019). Transarterial Infusion of iRGD-Modified ZrO(2) Nanoparticles with Lipiodol Improves the Tissue Distribution of Doxorubicin and Its Antitumor Efficacy. J. Vasc. Interv. Radiol..

[117] Simón-Gracia L., Hunt H., Scodeller P., Gaitzsch J., Kotamraju V.R., Sugahara K.N., Tammik O., Ruoslahti E., Battaglia G., Teesalu T. (2016). iRGD peptide conjugation potentiates intraperitoneal tumor delivery of paclitaxel with polymersomes. Biomaterials.

[118] Zhu Z., Xie C., Liu Q., Zhen X., Zheng X., Wu W., Li R., Ding Y., Jiang X., Liu B. (2011). The effect of hydrophilic chain length and iRGD on drug delivery from poly(ε-caprolactone)-poly(N-vinylpyrrolidone) nanoparticles. Biomaterials.

[119] Kang T., Li Y., Wang Y., Zhu J., Yang L., Huang Y., Xiong M., Liu J., Wang S., Huang M. (2019). Modular Engineering of Targeted Dual-Drug Nanoassemblies for Cancer Chemoimmunotherapy. ACS Appl. Mater. Interfaces.

[120] Liu C., Yao S., Li X., Wang F., Jiang Y. (2017). iRGD-mediated core-shell nanoparticles loading carmustine and O(6)-benzylguanine for glioma therapy. J. Drug Target..

[121] Bessone M.I.D., Simón-Gracia L., Scodeller P., de los Angeles R.M., Huvelle M.A.L., Soler-Illia G.J., Simian M. (2019). iRGD-guided tamoxifen polymersomes inhibit estrogen receptor transcriptional activity and decrease the number of breast cancer cells with self-renewing capacity. J. Nanobiotechnol..

[122] Gao F., Zhang J., Fu C., Xie X., Peng F., You J., Tang H., Wang Z., Li P., Chen J. (2017). iRGD-modified lipid-polymer hybrid nanoparticles loaded with isoliquiritigenin to enhance anti-breast cancer effect and tumor-targeting ability. Int. J. Nanomed..

[123] Lee J., Oh E.-T., Lee J., Kang T., Kim H.G., Kang H., Park H.J., Kim C. (2019). Cyclic iRGD peptide as a dual-functional on–off gatekeeper of mesoporous nanocontainers for targeting NRP-1 and selective drug release triggered by conformational conversion. New J. Chem..

[124] Su S., Wang H., Liu X., Wu Y., Nie G. (2013). iRGD-coupled responsive fluorescent nanogel for targeted drug delivery. Biomaterials.

[125] Zhang Y., Ma N., Luo C., Zhu J., Bao C. (2020). Photosensitizer-loaded cell membrane biomimetic nanoparticles for enhanced tumor synergetic targeted therapy. RSC Adv..

[126] Erel-Akbaba G., Carvalho L.A., Tian T., Zinter M., Akbaba H., Obeid P.J., Chiocca E.A., Weissleder R., Kantarci A.G., Tannous B.A. (2019). Radiation-Induced Targeted Nanoparticle-Based Gene Delivery for Brain Tumor Therapy. ACS Nano.

[127] Jin Z., Lv Y., Cao H., Yao J., Zhou J., He W., Yin L. (2016). Core-shell nanocarriers with high paclitaxel loading for passive and active targeting. Sci. Rep..

[128] Gu G., Gao X., Hu Q., Kang T., Liu Z., Jiang M., Miao D., Song Q., Yao L., Tu Y. (2013). The influence of the penetrating peptide iRGD on the effect of paclitaxel-loaded MT1-AF7p-conjugated nanoparticles on glioma cells. Biomaterials.

[129] Zhong Y., Su T., Shi Q., Feng Y., Tao Z., Huang Q., Li L., Hu L., Li S., Tan H. (2019). Co-Administration of iRGD Enhances Tumor-Targeted Delivery and Anti-Tumor Effects of Paclitaxel-Loaded PLGA Nanoparticles for Colorectal Cancer Treatment. Int. J. Nanomed..

[130] Deng C., Xu X., Tashi D., Wu Y., Su B., Zhang Q. (2018). Co-administration of biocompatible self-assembled polylactic acid-hyaluronic acid block copolymer nanoparticles with tumor-penetrating peptide-iRGD for metastatic breast cancer therapy. J. Mater. Chem. B.

[131] Liu X., Lin P., Perrett I., Lin J., Liao Y.P., Chang C.H., Jiang J., Wu N., Donahue T., Wainberg Z. (2017). Tumor-penetrating peptide enhances transcytosis of silicasome-based chemotherapy for pancreatic cancer. J. Clin. Investig..

[132] Deng C., Zhang Q., Fu Y., Sun X., Gong T., Zhang Z. (2017). Coadministration of Oligomeric Hyaluronic Acid-Modified Liposomes with Tumor-Penetrating Peptide-iRGD Enhances the Antitumor Efficacy of Doxorubicin against Melanoma. ACS Appl. Mater. Interfaces.

[133] Yu H., Tang Z., Song W., Zhang D., Zhang Y., Chen X. (2015). Co-administration of iRGD enhancing the anticancer efficacy of cisplatin-loaded polypeptide nanoparticles. J. Control. Release.

[134] Cun X., Chen J., Ruan S., Zhang L., Wan J., He Q., Gao H. (2015). A Novel Strategy through Combining iRGD Peptide with Tumor-Microenvironment-Responsive and Multistage Nanoparticles for Deep Tumor Penetration. ACS Appl. Mater. Interfaces.

[135] Umeshappa C.S., Shao K. (2018). Comment on “Coadministration of iRGD with Multistage Responsive Nanoparticles Enhanced Tumor Targeting and Penetration Abilities for Breast Cancer Therapy”. ACS Appl. Mater. Interfaces.

[136] Zhang Q., Zhang Y., Li K., Wang H., Li H., Zheng J. (2015). A Novel Strategy to Improve the Therapeutic Efficacy of Gemcitabine for Non-Small Cell Lung Cancer by the Tumor-Penetrating Peptide iRGD. PLoS ONE.

[137] Zhang Y., Yang J., Ding M., Li L., Lu Z., Zhang Q., Zheng J. (2016). Tumor-penetration and antitumor efficacy of cetuximab are enhanced by co-administered iRGD in a murine model of human NSCLC. Oncol. Lett..

[138] Fadeev R., Chekanov A., Solovieva M., Bezborodova O., Nemtsova E., Dolgikh N., Fadeeva I., Senotov A., Kobyakova M., Evstratova Y. (2019). Improved Anticancer Effect of Recombinant Protein izTRAIL Combined with Sorafenib and Peptide iRGD. Int. J. Mol. Sci..

[139] Zhang D., Chu Y., Qian H., Qian L., Shao J., Xu Q., Yu L., Li R., Zhang Q., Wu F. (2020). Antitumor Activity of Thermosensitive Hydrogels Packaging Gambogic Acid Nanoparticles and Tumor-Penetrating Peptide iRGD Against Gastric Cancer. Int. J. Nanomed..

[140] Saad A.M., Turk T., Al-Husseini M.J., Abdel-Rahman O. (2018). Trends in pancreatic adenocarcinoma incidence and mortality in the United States in the last four decades; a SEER-based study. BMC Cancer.

[141] Khalaf N., El-Serag H.B., Abrams H.R., Thrift A.P. (2020). Burden of Pancreatic Cancer—From Epidemiology to Practice. Clin. Gastroenterol. Hepatol..

[142] Rahib L., Smith B.D., Aizenberg R., Rosenzweig A.B., Fleshman J.M., Matrisian L.M. (2014). Projecting cancer incidence and deaths to 2030: The unexpected burden of thyroid, liver, and pancreas cancers in the United States. Cancer Res..

[143] Oberstein P.E., Olive K.P. (2013). Pancreatic cancer: Why is it so hard to treat?. Ther. Adv. Gastroenterol..

[144] Conroy T., Desseigne F., Ychou M., Bouche O., Guimbaud R., Becouarn Y., Adenis A., Raoul J.L., Gourgou-Bourgade S., de la Fouchardiere C. (2011). FOLFIRINOX versus gemcitabine for metastatic pancreatic cancer. N. Engl. J. Med..

[145] Frese K.K., Neesse A., Cook N., Bapiro T.E., Lolkema M.P., Jodrell D.I., Tuveson D.A. (2012). Nab-Paclitaxel potentiates gemcitabine activity by reducing cytidine deaminase levels in a mouse model of pancreatic cancer. Cancer Discov..

[146] Von Hoff D.D., Ramanathan R.K., Borad M.J., Laheru D.A., Smith L.S., Wood T.E., Korn R.L., Desai N., Trieu V., Iglesias J.L. (2011). Gemcitabine plus nab-paclitaxel is an active regimen in patients with advanced pancreatic cancer: A phase I/II trial. J. Clin. Oncol..

[147] Fukahi K., Fukasawa M., Neufeld G., Itakura J., Korc M. (2004). Aberrant expression of neuropilin-1 and -2 in human pancreatic cancer cells. Clin. Cancer Res..

[148] Wey J.S., Gray M.J., Fan F., Belcheva A., McCarty M.F., Stoeltzing O., Somcio R., Liu W., Evans D.B., Klagsbrun M. (2005). Overexpression of neuropilin-1 promotes constitutive MAPK signalling and chemoresistance in pancreatic cancer cells. Br. J. Cancer.

[149] Fukasawa M., Matsushita A., Korc M. (2007). Neuropilin-1 interacts with integrin beta1 and modulates pancreatic cancer cell growth, survival and invasion. Cancer Biol. Ther..

[150] Matkar P.N., Singh K.K., Rudenko D., Kim Y.J., Kuliszewski M.A., Prud’homme G.J., Hedley D.W., Leong-Poi H. (2016). Novel regulatory role of neuropilin-1 in endothelial-to-mesenchymal transition and fibrosis in pancreatic ductal adenocarcinoma. Oncotarget.

[151] DrugCendR CEND-1 in Combination with Nabpaclitaxel and Gemcitabine in Metastatic Pancreatic Cancer. https://clinicaltrials.gov/ct2/show/NCT03517176.

[152] Slaney C.Y., Kershaw M.H., Darcy P.K. (2014). Trafficking of T cells into tumors. Cancer Res..

[153] Sun Y., Wang H., Wang P., Zhang K., Geng X., Liu Q., Wang X. (2019). Tumor targeting DVDMS-nanoliposomes for an enhanced sonodynamic therapy of gliomas. Biomater. Sci..

[154] Martin M., Paul D., Orwin M., Schlievert P. (2000). Exotoxins of *Staphylococcus aureus*. Clin. Microbiol. Rev..

[155] Song Y., Xu M., Li Y., Li Y., Gu W., Halimu G., Fu X., Zhang H., Zhang C. (2020). An iRGD peptide fused superantigen mutant induced tumor-targeting and T lymphocyte infiltrating in cancer immunotherapy. Int. J. Pharm..

[156] Ricci M.S., Zong W.-X. (2006). Chemotherapeutic approaches for targeting cell death pathways. Oncologist.

[157] Li Y., Ahmed F., Ali S., Philip P.A., Kucuk O., Sarkar F.H. (2005). Inactivation of nuclear factor κB by soy isoflavone genistein contributes to increased apoptosis induced by chemotherapeutic agents in human cancer cells. Cancer Res..

[158] Dudek-Perić A.M., Ferreira G.B., Muchowicz A., Wouters J., Prada N., Martin S., Kiviluoto S., Winiarska M., Boon L., Mathieu C. (2015). Antitumor immunity triggered by melphalan is potentiated by melanoma cell surface–associated calreticulin. Cancer Res..

[159] Zitvogel L., Apetoh L., Ghiringhelli F., Kroemer G. (2008). Immunological aspects of cancer chemotherapy. Nat. Rev. Immunol..

[160] Casares N., Pequignot M.O., Tesniere A., Ghiringhelli F., Roux S., Chaput N., Schmitt E., Hamai A., Hervas-Stubbs S., Obeid M. (2005). Caspase-dependent immunogenicity of doxorubicin-induced tumor cell death. J. Exp. Med..

[161] Ding Z.C., Munn D.H., Zhou G. (2014). Chemotherapy-induced myeloid suppressor cells and antitumor immunity: The Janus face of chemotherapy in immunomodulation. Oncoimmunology.

[162] Pruneri G., Gray K.P., Vingiani A., Viale G., Curigliano G., Criscitiello C., Láng I., Ruhstaller T., Gianni L., Goldhirsch A. (2016). Tumor-infiltrating lymphocytes (TILs) are a powerful prognostic marker in patients with triple-negative breast cancer enrolled in the IBCSG phase III randomized clinical trial 22-00. Breast Cancer Res. Treat..

[163] Lake R.A., Robinson B.W. (2005). Immunotherapy and chemotherapy—A practical partnership. Nat. Rev. Cancer.

[164] Chang C.L., Hsu Y.T., Wu C.C., Lai Y.Z., Wang C., Yang Y.C., Wu T.C., Hung C.F. (2013). Dose-dense chemotherapy improves mechanisms of antitumor immune response. Cancer Res..

[165] Deng C., Jia M., Wei G., Tan T., Fu Y., Gao H., Sun X., Zhang Q., Gong T., Zhang Z. (2017). Inducing Optimal Antitumor Immune Response through Coadministering iRGD with Pirarubicin Loaded Nanostructured Lipid Carriers for Breast Cancer Therapy. Mol. Pharm..

[166] Kolishetti N., Dhar S., Valencia P.M., Lin L.Q., Karnik R., Lippard S.J., Langer R., Farokhzad O.C. (2010). Engineering of self-assembled nanoparticle platform for precisely controlled combination drug therapy. Proc. Natl. Acad. Sci. USA.

[167] Ferrari M. (2005). Cancer nanotechnology: Opportunities and challenges. Nat. Rev. Cancer.

[168] Han W., Shi L., Ren L., Zhou L., Li T., Qiao Y., Wang H. (2018). A nanomedicine approach enables co-delivery of cyclosporin A and gefitinib to potentiate the therapeutic efficacy in drug-resistant lung cancer. Signal. Transduct Target. Ther..

[169] Le Q.-V., Choi J., Oh Y.-K. (2018). Nano delivery systems and cancer immunotherapy. J. Pharm. Investig..

[170] Tran P., Lee S.-E., Kim D.-H., Pyo Y.-C., Park J.-S. (2020). Recent advances of nanotechnology for the delivery of anticancer drugs for breast cancer treatment. J. Pharm. Investig..

[171] Park Y.J., Kuen D.S., Chung Y. (2018). Future prospects of immune checkpoint blockade in cancer: From response prediction to overcoming resistance. Exp. Mol. Med..

[172] Kunimasa K., Goto T. (2020). Immunosurveillance and Immunoediting of Lung Cancer: Current Perspectives and Challenges. Int. J. Mol. Sci..

[173] Kavanagh B., O’Brien S., Lee D., Hou Y., Weinberg V., Rini B., Allison J.P., Small E.J., Fong L. (2008). CTLA4 blockade expands FoxP3+ regulatory and activated effector CD4+ T cells in a dose-dependent fashion. Blood.

[174] Schwartz R.H. (1992). Costimulation of T lymphocytes: The role of CD28, CTLA-4, and B7/BB1 in interleukin-2 production and immunotherapy. Cell.

[175] Linsley P.S., Brady W., Urnes M., Grosmaire L.S., Damle N.K., Ledbetter J.A. (1991). CTLA-4 is a second receptor for the B cell activation antigen B7. J. Exp. Med..

[176] Schneider H., Downey J., Smith A., Zinselmeyer B.H., Rush C., Brewer J.M., Wei B., Hogg N., Garside P., Rudd C.E. (2006). Reversal of the TCR stop signal by CTLA-4. Science.

[177] Krummel M.F., Allison J.P. (1995). CD28 and CTLA-4 have opposing effects on the response of T cells to stimulation. J. Exp. Med..

[178] Walunas T.L., Lenschow D.J., Bakker C.Y., Linsley P.S., Freeman G.J., Green J.M., Thompson C.B., Bluestone J.A. (1994). CTLA-4 can function as a negative regulator of T cell activation. Immunity.

[179] Kwiecien I., Stelmaszczyk-Emmel A., Polubiec-Kownacka M., Dziedzic D., Domagala-Kulawik J. (2017). Elevated regulatory T cells, surface and intracellular CTLA-4 expression and interleukin-17 in the lung cancer microenvironment in humans. Cancer Immunol. Immunother..

[180] Chemnitz J.M., Parry R.V., Nichols K.E., June C.H., Riley J.L. (2004). SHP-1 and SHP-2 associate with immunoreceptor tyrosine-based switch motif of programmed death 1 upon primary human T cell stimulation, but only receptor ligation prevents T cell activation. J. Immunol..

[181] Parry R.V., Chemnitz J.M., Frauwirth K.A., Lanfranco A.R., Braunstein I., Kobayashi S.V., Linsley P.S., Thompson C.B., Riley J.L. (2005). CTLA-4 and PD-1 receptors inhibit T-cell activation by distinct mechanisms. Mol. Cell Biol..

[182] Keir M.E., Butte M.J., Freeman G.J., Sharpe A.H. (2008). PD-1 and its ligands in tolerance and immunity. Annu. Rev. Immunol..

[183] Chen J., Jiang C.C., Jin L., Zhang X.D. (2016). Regulation of PD-L1: A novel role of pro-survival signalling in cancer. Ann. Oncol..

[184] Rosenberg J.E., Hoffman-Censits J., Powles T., van der Heijden M.S., Balar A.V., Necchi A., Dawson N., O’Donnell P.H., Balmanoukian A., Loriot Y. (2016). Atezolizumab in patients with locally advanced and metastatic urothelial carcinoma who have progressed following treatment with platinum-based chemotherapy: A single-arm, multicentre, phase 2 trial. Lancet.

[185] Pardoll D.M. (2012). The blockade of immune checkpoints in cancer immunotherapy. Nat. Rev. Cancer.

[186] Wei S.C., Duffy C.R., Allison J.P. (2018). Fundamental Mechanisms of Immune Checkpoint Blockade Therapy. Cancer Discov..

[187] Yang J., Hu L. (2019). Immunomodulators targeting the PD-1/PD-L1 protein-protein interaction: From antibodies to small molecules. Med. Res. Rev..

[188] Seidel J.A., Otsuka A., Kabashima K. (2018). Anti-PD-1 and anti-CTLA-4 therapies in cancer: Mechanisms of action, efficacy, and limitations. Front. Oncol..

[189] Yang J., Yang J., Wei Y., Yin H., Fang L., Chai D., Li H., Li H., Zhang Q., Zheng J. (2019). Modification of IL-24 by tumor penetrating peptide iRGD enhanced its antitumor efficacy against non-small cell lung cancer. Int. Immunopharmacol..

[190] Jarvelainen H., Botta G.P., De Mendoza T.H., Ruoslahti E. Co-administration of the iRGD tumor-penetrating peptide improves the tumor immunostimulatory effects of low-dose IL-2. Proceedings of the AACR Annual Meeting.

[191] Zhang F., Stephan S.B., Ene C.I., Smith T.T., Holland E.C., Stephan M.T. (2018). Nanoparticles That Reshape the Tumor Milieu Create a Therapeutic Window for Effective T-cell Therapy in Solid Malignancies. Cancer Res..

[192] Ding N., Zou Z., Sha H., Su S., Qian H., Meng F., Chen F., Du S., Zhou S., Chen H. (2019). iRGD synergizes with PD-1 knockout immunotherapy by enhancing lymphocyte infiltration in gastric cancer. Nat. Commun..

[193] Zhao M., van Straten D., Broekman M.L.D., Preat V., Schiffelers R.M. (2020). Nanocarrier-based drug combination therapy for glioblastoma. Theranostics.

[194] Rubin L.L., Staddon J.M. (1999). The cell biology of the blood-brain barrier. Annu. Rev. Neurosci..

[195] Luissint A.C., Federici C., Guillonneau F., Chretien F., Camoin L., Glacial F., Ganeshamoorthy K., Couraud P.O. (2012). Guanine nucleotide-binding protein Galphai2: A new partner of claudin-5 that regulates tight junction integrity in human brain endothelial cells. J. Cereb. Blood Flow Metab..

[196] Pardridge W.M. (2012). Drug transport across the blood-brain barrier. J. Cereb. Blood Flow Metab..

[197] Nishio S., Ohta M., Abe M., Kitamura K. (1983). Microvascular abnormalities in ethylnitrosourea (ENU)-induced rat brain tumors: Structural basis for altered blood-brain barrier function. Acta Neuropathol..

[198] Wolburg H., Noell S., Fallier-Becker P., Mack A.F., Wolburg-Buchholz K. (2012). The disturbed blood-brain barrier in human glioblastoma. Mol. Asp. Med..

[199] Gottesman M.M., Fojo T., Bates S.E. (2002). Multidrug resistance in cancer: Role of ATP-dependent transporters. Nat. Rev. Cancer.

[200] Li Y., Yuan H., Yang K., Xu W., Tang W., Li X. (2010). The structure and functions of P-glycoprotein. Curr. Med. Chem..

[201] Siegal T., Rubinstein R., Bokstein F., Schwartz A., Lossos A., Shalom E., Chisin R., Gomori J.M. (2000). In vivo assessment of the window of barrier opening after osmotic blood-brain barrier disruption in humans. J Neurosurg..

[202] Van Tellingen O., Yetkin-Arik B., de Gooijer M.C., Wesseling P., Wurdinger T., de Vries H.E. (2015). Overcoming the blood-brain tumor barrier for effective glioblastoma treatment. Drug Resist. Updates.

[203] Raymond J.J., Robertson D.M., Dinsdale H.B. (1986). Pharmacological modification of bradykinin induced breakdown of the blood-brain barrier. Can. J. Neurol. Sci..

[204] Lu C.T., Zhao Y.Z., Wong H.L., Cai J., Peng L., Tian X.Q. (2014). Current approaches to enhance CNS delivery of drugs across the brain barriers. Int. J. Nanomed..

[205] Vogelbaum M.A., Aghi M.K. (2015). Convection-enhanced delivery for the treatment of glioblastoma. Neuro-Oncology.

[206] Hamilton A.M., Aidoudi-Ahmed S., Sharma S., Kotamraju V.R., Foster P.J., Sugahara K.N., Ruoslahti E., Rutt B.K. (2015). Nanoparticles coated with the tumor-penetrating peptide iRGD reduce experimental breast cancer metastasis in the brain. J. Mol. Med..

[207] Moncelet D., Bouchaud V., Mellet P., Ribot E., Miraux S., Franconi J.M., Voisin P. (2013). Cellular density effect on RGD ligand internalization in glioblastoma for MRI application. PLoS ONE.

[208] Liu X., Li H., Jin Q., Ji J. (2014). Surface tailoring of nanoparticles via mixed-charge monolayers and their biomedical applications. Small.

[209] Ruoslahti E. (2012). Peptides as targeting elements and tissue penetration devices for nanoparticles. Adv. Mater..

